# Moonlighting genes harbor antisense ORFs that encode potential membrane proteins

**DOI:** 10.1038/s41598-023-39869-x

**Published:** 2023-08-03

**Authors:** Kasman E. Thomas, Paul A. Gagniuc, Elvira Gagniuc

**Affiliations:** 1 Synevovet Laboratory, Bucharest, Romania; 2 Faculty of Engineering in Foreign Languages, University Politehnica of Bucharest, Bucharest, Romania; 3 Faculty of Veterinary Medicine, University of Agronomic Sciences and Veterinary Medicine, Bucharest, Romania

**Keywords:** Gene delivery, Genomics, Computational models, Data mining, Software, Statistical methods

## Abstract

Moonlighting genes encode for single polypeptide molecules that perform multiple and often unrelated functions. These genes occur across all domains of life. Their ubiquity and functional diversity raise many questions as to their origins, evolution, and role in the cell cycle. In this study, we present a simple bioinformatics probe that allows us to rank genes by antisense translation potential, and we show that this probe enriches, reliably, for moonlighting genes across a variety of organisms. We find that moonlighting genes harbor putative antisense open reading frames (ORFs) rich in codons for non-polar amino acids. We also find that moonlighting genes tend to co-locate with genes involved in cell wall, cell membrane, or cell envelope production. On the basis of this and other findings, we offer a model in which we propose that moonlighting gene products are likely to escape the cell through gaps in the cell wall and membrane, at wall/membrane construction sites; and we propose that antisense ORFs produce “membrane-sticky” protein products, effectively binding moonlighting-gene DNA to the cell membrane in porous areas where intensive cell-wall/cell-membrane construction is underway. This leads to high potential for escape of moonlighting proteins to the cell surface. Evolutionary and other implications of these findings are discussed.

## Introduction

Moonlighting genes are genes that encode proteins having multiple distinct and often unrelated functions^1^. Paradoxically, these proteins often have a cytosolic location as well as being found on the exterior of the cell. To our knowledge, no secretion-system partners have been identified for these proteins. In the 30 years since glyceraldehyde 3-phosphate dehydrogenase (GAPDH) was found to have a secondary role on the cell surface of pathogenic streptococci^2^, many other examples of moonlighting have been uncovered. Such examples include gene products with well-known cytosolic roles that somehow end up on the surface of the cell, or are excreted into culture media. The manually curated MoonProt database now lists over 300 such genes, spanning host organisms that range from bacteria to yeast, protists, archeons, plants, and mammals. Many fundamental questions remain unanswered: How do these genes acquire multiple functions? How do they gain access to the exterior of the cell, in the absence of secretion-system partners? Why do some metabolic enzymes get secreted while many others do not? And how is it that the same proteins (e.g. GAPDH, enolase, DnaK, GroEL, Ef-Tu, superoxide dismutase) serve in moonlighting roles across diverse hosts? Because the same proteins are often found in moonlighting roles across phyla, it seems likely that the phenomenon is made possible by processes that are fundamental to all life. Of note is that many genes involved in moonlighting are ancient, highly conserved genes, again pointing to underlying processes that are fundamental—perhaps even primordial, in some sense. In the present study, we aim for a top-down bioinformatics investigation of moonlighting genes, in which we look for high-level clues and pan-genomic causes and effects.

### High-level overview

A key characteristic of cellular life is encapsulation: cells have an inside, and an outside, with durable structures separating the two. One way of looking at it is that the cell embodies an entropy gradient, with a high-entropy aqueous environment in the center, and a low-entropy (which is to say, highly structured) envelope, encompassing a membrane and structural components, at the periphery. The membrane components of the cell are largely composed of proteins containing non-polar amino acids; whereas by contrast, water-soluble proteins (such as those present at the center of the cell) have mostly polar amino acids on their surface. The genetic code offers a convenient (and universal) mechanism for specifying polar versus non-polar amino acids: a purine at base two of a codon virtually guarantees the selection of a polar amino acid, while a pyrimidine in the second base tends to guarantee a non-polar amino acid. This suggests a primordial genetic code that may (arguably, at least) have been a binary code, allowing either for polar or non-polar amino acids, based on the use of purines or pyrimidines in codons. (For discussion of this possibility, see Trifonov^3^). Whether RNA or DNA, the primordial genetic material may have been single-stranded, in which case transcription to mRNA (if it occurred) could happen in one direction only, namely in a 3′-to-5′ manner. However, with the arrival of double-stranded nucleic acids, transcription could occur in either of two directions. Due to complementarity, a message that encodes polar amino acids in one direction would naturally tend to encode non-polar amino acids in the other direction. A scenario can be imagined in the early days of double-stranded genetic material, namely the days before promoters, repressors, Shine Dalgarno sequences or other specialized sequence organizations such as UTRs/non-coding regions: at that point in time, transcription may have occurred bidirectionally, with water-soluble proteins produced in one direction and proteins rich in hydrophobic amino acids produced in the other direction, a situation that leads quite naturally to the production of membrane proteins and encapsulation of hydrophilic proteins within membranes (i.e., cellular life). Moonlighting is largely an issue involving “inside versus outside.” Therefore, it is only natural to wonder if clues to the phenomenon might involve questions of hydrophobic amino acid usage, and/or cell wall and cell membrane construction. We consider this and other questions in formulating bioinformatic techniques designed to discover moonlighting genes.

## Materials and methods

Our model organisms include *Streptococcus pneumoniae* NCTC11032 (G + C content 40.6%), *Escherichia coli* NCTC11775 (G + C 51.7%), and *Mycobacterium tuberculosis* H37Rv (G + C 65.9%). RefSeq genomes were downloaded from NCBI’s repository. A total of 25 moonlighting genes were curated from the MoonProt database. These are genes for which ample evidence exists of moonlighting activity (Table 1). Many of these genes exist in more than one isoform. For our enrichment experiments, we count each isoform separately. For example, in *M. tuberculosis*, cysteine desulfurase exists as genes *csd* and *iscS*; superoxide dismutase exists as *SodA* and *sodC*; and so on. Altogether, counting all isoforms of all genes, *M. tuberculosis* contains 35 moonlighting-protein genes; *E. coli* was found to have 31; *S. pneumoniae* has 20.

**Table 1:** Targeted moonlighting genes.

Gene name	Primary function	Secondary function	Organism(s)	Refs.
Methyltransferase Erm	Methylation of the 23S rRNA at A2058	Dimethylates arginine 42 of histone H3 in host cells	*Mycobacterium tuberculosis*	^15^
Glutamate racemase	Cell wall biogenesis, peptidoglycan biosynthesis	DNA gyrase inhibitor	*Mycobacterium tuberculosis*	^16,17^
Elongation factor Tu	Translation elongation factor	Plasminogen binding	*Bifidobacterium longum, Lactobacillus johnsonii, Mycoplasma pneumoniae, Streptococcus gordonii, Candida albicans, Homo sapiens*	^18^
Malate synthase	Carbohydrate metabolism; glyoxylate cycle	Binds fibronectin, laminin, and A549 lung epithelial cells	*Mycobacterium tuberculosis*	^19^
Cysteine desulfurase	Conversion of l-cysteine to l-alanine and sulfane sulfur	Found on cell surface of Mycobacterium	*Mycobacterium tuberculosis*	^20^
Gamma-glutamyl phosphate reductase	ProA. Catalyzes second reaction in production of proline from glutamate	Found on cell surface of Mycobacterium	*Mycobacterium tuberculosis*	^21^
Glucose-6-phosphate isomerase	Interconversion of d-glucose 6-phosphate and d-fructose 6-phosphate in glycolysis	Laminin, collagen I binding	*Staphylococcus aureus, Lactobacillus crispatus, Candida albicans*	^1^
6-Phosphofructokinase	Phosphorylates fructose 6-phosphate in glycolysis	Binds plasminogen	*Lactococcus lactis, Streptococcus oralis, Pichia pastoris, Homo sapiens*	^22,19,23^
6-Phosphogluconate dehydrogenase	Carbohydrate degradation, pentose phosphate pathway	Adhesin that induces immune response in mice	*Streptococcus pneumoniae, Candida albicans*	^24^
Enolase	Converts 2-phospho-d-glycerate to phosphoenolpyruvate in glycolysis	Binds plasminogen, fibronectin, and laminin	*Aeromonas hydrophila, Bifidobacterium longum, Borrelia burgdorferi, Lactobacillus crispatus, Neisseria meningitidis, Staphylococcus aureus, numerous others*	^25–27^
triose-phosphate isomerase	Catalyzes the interconversion of dihydroxyacetone phosphate (DHAP) and glyceraldehyde 3-phosphate	Plasminogen binding	*Paracoccidioides brasiliensis, Staphylococcus aureus, Streptococcus oralis*	^28^
fusA	Translation elongation factor G (EF-G)	Adhesin, binds salivary mucin MUC7	*Streptococcus gordonii*	^29^
pepO	Endopeptidase O	Binds plasminogen and fibronectin. Regulates SpeB expression	*Streptococcus pneumoniae*	^2^
rpoB	DNA-directed RNA polymerase beta subunit	Muc7 binding protein	*Streptococcus gordonii*	^30^
DnaK	Heat shock 70 kDa protein	Binds plasminogen	*Bifidobacterium longum, Mycobacterium tuberculosis, Neisseria meningitidis, Lactococcus lactis*	^31,32,15^
GroEL	Chaperone, aids protein folding	Adhesin, binds mucins and to CD43 on macrophage surface	*Listeria monocytogenes, Chlamydiae pneumoniae, Legionella pneumophila, Haemophilus ducreyi, Lactobacillus johnsonii, Salmonella typhimurium, Clostridium difficile, Helicobacter pylori , M. tuberculosis*	^33–38^, ^15^
Diacylglycerol acyltransferase/mycolyltransferase A85A	Transesterification of mycolic acids	Binds fibronectin	*Mycobacterium tuberculosis*	^39^
Diacylglycerol acyltransferase/mycolyltransferase A85B	Transesterification of mycolic acids	Binds fibronectin	*Mycobacterium tuberculosis*	^40^
Diacylglycerol acyltransferase/mycolyltransferase A85C	Transesterification of mycolic acids	Binds fibronectin	*Mycobacterium tuberculosis*	^20^
Superoxide dismutase	Conversions of superoxide anion radicals into O2 and H2O2	Adhesin	*Mycobacterium avium*	^21^
Glyceraldehyde.3-phosphate dehydrogenase	Conversion of glyceraldehyde 3-phosphate to D-glycerate 1,3-bisphosphate	Binds fibronectin, laminin, type I collagen, mucin, and Caco-2 cells	*Paracoccidioides brasiliensis, Streptococcus pyogenes, Staphylococcus aureus, Bacillus anthracis, Mycoplasma genitalium; yeast, fungi, worms, mammals*	^41,19^, ^28,42^
Phosphoglycerate kinase	Production of 3-phosphoglycerate and ATP from 1,3-bisphosphoglycerate and ADP	Binds plasminogen	*Bifidobacterium longum, Streptococcus agalactiae, Candida albicans, Homo sapiens*	^43^
Fructose-bisphosphate aldolase	Conversion of D-fructose 1,6-bisphosphate to dihydroxyacetone phosphate and D-glyceraldehyde 3-phosphate	Adhesin, binds Flamingo cadherin receptor (FCR); binds fibronectin. In Plasmodium berghei, attaches actin filaments to TRAP proteins (transmembrane adhesive proteins of the thrombospondin-related anonymous protein) and transduces the motor force across the surface of the plasmodium	*Streptococcus pneumoniae, Candida tropicalis, Plasmodium berghei, Toxoplasma gondii, Mus musculus, Homo sapiens, others*	^44–46^
Phosphoglycerate mutase	Interconversion of 1,3-bisphosphoglycerate and 2,3-bisphosphoglycerate	Binds plasminogen	*Bifidobacterium lactis, Streptococcus oralis,Candida albicans*	^12,45,28^
Glutamine synthetase	Conversion of glutamate to glutamine	Binds plasminogen	*Bifidobacterium lactis, Lactobacillus crispatus, Mycobacterium tuberculosis, Bacillus subtilis*	^31^

A total of 25 genes were curated from the MoonProt database. The fourth column shows the names of some organisms for which the Secondary Function has been documented. Note that in a given host, genes can exist in more than one isoform, and/or as subunits. For example, in *M. tuberculosis*, there are two isoforms of Elongation Factor G, while glutamine synthetase has four subunits. In the enrichment experiments each isoform is considered a separate gene.

The genes selected for this study have in common the characteristic that all are known to produce proteins having a cytosolic location as well as an extra-cytosolic location (either on the surface of the cell, or excreted into culture medium). Thus, they qualify under the rubric that has been called “Excretion of Cytosolic Proteins,” or ECP^4^.

We designed an enrichment assay in which we first score every gene on the basis of a metric, then sort genes by their scores, then obtain the top-scoring 20% of all genes. Within that top cut, we look for moonlighting genes, and other functional categories of genes. We then calculate fold-enrichment numbers, and compute an expectation value for each one based on cumulative hypergeometric probability. (The code for the enrichment analysis as well as for the hypergeometric-probability analysis is freely available at https://github.com/kasmanethomas/moonlighting/tree/main). We tried various “cur sizes” from 5 to 30% and consistently found enrichments at all cutpoints. The fold enrichments tended to be higher at smaller sample sizes. We settled on 20% as a cut size that would be appropriately inclusive yet not overly broad. The somewhat lower fold enrichments seen at this cut size mean that the numbers are properly conservative.

The metrics we use involve tallying the number (as a percent) of codons meeting a certain description: for example, one metric tallies the percentage of codons that match the pattern RNY, where ‘R’ is any purine, ‘N’ is any base, and ‘Y’ is any pyrimidine. Another metric we use involves obtaining Shannon entropies for purines/pyrimidines in bases one and three of all of a gene’s codons; these two entropies are then used to construct a 2D vector. Likewise we obtain the G + C entropies of bases one and three for a gene’s codons; these two entropies form a 2D vector. A metric is derived from the dot product of the two 2D vectors. (The motivation behind this metric is discussed in “Results”).

The code for calculating metrics and doing the enrichment assays consists of native JavaScript code created by the authors (see the Github repository at https://github.com/kasmanethomas/moonlighting for code listings). Our code conforms to ECMAScript2015 and we tested it in Google Chrome Version 107.0.5304.121 (x86_64).

## Results

Our investigation revealed that two common factors exist for all moonlighting genes: first, they tend to be physically located near genes for enzymes involved in cell wall, cell membrane, or cell envelope construction; and second, they tend to encode, in antisense, small proteins that contain a high percentage of non-polar amino acids. We began by looking at where moonlighting genes occur on the genome of each biological model and found that they tend to co-locate with genes involved in cell wall and cell membrane construction. We then characterized moonlighting genes with respect to codon purine bias—and found that moonlighting genes have higher-than-average purine bias in both forward and backward (reverse complement) directions. Next, we developed enrichment assays based on these codon characteristics. The results of those assays were consistent with the idea that antisense open reading frames might exist in moonlighting genes. Accordingly, we searched for antisense ORFs in moonlighting genes. We found that not only do such ORFs exist, they often contain predicted transmembrane domains.

### Location of moonlighting genes

In order to get an idea of the local environment in which moonlighting genes “operate,” we looked at their proximity to other genes. We asked: what are their neighbors? We can make a simple experiment of sampling for all genes that lie within plus or minus a certain distance (say five genes) of moonlighting genes, being careful to remove duplicate hits. The set of all nearest-neighbors within five genes of a moonlighting gene was tested. After the removal of duplicates, in *M. tuberculosis* H37Rv we found 298 genes (N = 298) with enrichment characteristics as shown in Table 2. Note: Similar results were found with a proximity radius of three as well as with ten. A radius of five was chosen because at lower ranges, the result set was comparatively sparse, containing only 136 genes, whereas at higher ranges, fold-enrichments tended to be low, with higher E-values. The most informative result-set was obtained at a radius of five.

**Table 2:** Enrichment: neighbor genes (N = 304) within 5 genes of moonlighting genes in *M. tuberculosis* H37Rv.

Fold	E	Function	Found/existing
1.48	0.190	Cell wall biogenesis	7/62
1.23	0.362	Secretion	6/64
1.19	0.582	Inner membrane	1/11
1.17	0.349	Fatty acid	10/112
1.10	0.162	Hypothetical protein	88/1052

Fold enrichments and hypergeometric expectation values for the N = 298 genes in close proximity to moonlighting genes of *Mycobacterium tuberculosis* H37Rv. Categories are based on Gene Ontology ensembles (see “Materials and methods”).

Notice that moonlighting genes tend to co-locate with cell-wall biogenesis genes. However, the most important numerical result is the large number of “hypothetical protein” genes found (Table 2). Almost 30% of the search-result set is composed of hypothetical-protein genes. It turns out, a much more informative picture can be seen once the “hypothetical protein” genes are no longer diluting our results. If we filter out the hypothetical-protein genes on the basis that they may be obscuring hidden results, and count only genes for which a function has been assigned, the enrichments look as shown in Table 3.

**Table 3:** Enrichment: neighbor genes (N = 210) within 5 genes of moonlighting genes in *M. tuberculosis* H37Rv, with “hypothetical protein” genes filtered.

Fold	E	Function	Found/existing
2.10	0.046	Cell wall biogenesis	7/62
1.74	0.127	Secretion	6/64
1.69	0.455	Inner membrane	1/11
1.66	0.076	Fatty acid	10/112
1.43	0.305	Peptidoglycan	4/52
1.33	0.449	Lipoprotein	2/28
1.33	0.355	Outer membrane	4/56
1.10	0.402	Plasma membrane	12/202
1.09	0.609	tRNA ligase	1/17

Fold enrichments and hypergeometric expectation values for the N = 210 genes in close proximity to moonlighting genes of Mycobacterium tuberculosis H37Rv.

Notice that genes involved in cell wall biogenesis, secretion, or inner membrane function are at the top of the list. However, the list now also includes many other important categories, including outer membrane, plasma membrane, and genes specifically involved in peptidoglycan synthesis. Similar results are obtained in *E. coli* (Table 4) and *Streptococcus pneumoniae* (please see Table 5).

**Table 4:** Enrichment: neighbor genes (N = 294) within 5 genes of moonlighting genes in *E. coli* NCTC11775, with “hypothetical protein” genes filtered.

Fold	E	Function	Found/existing
2.70	0.021	Exporter	6/36
2.50	0.001	Secretion	13/84
2.11	0.010	Peptidoglycan	12/92
1.73	0.248	tRNA ligase	3/28
1.58	0.245	Inner membrane	4/41
1.28	0.419	Lipoprotein	3/38
1.24	0.172	Outer membrane	21/273

**Table 5:** Enrichment: neighbor genes (N = 176) within 5 genes of moonlighting genes in *S. pneumoniae* NCTC11032, with “hypothetical protein” genes filtered.

Fold	E	Function	Found/existing
2.83	0.309	Cell division	1/4
2.83	0.309	Peptidoglycan	1/4
2.26	0.370	Inner membrane	1/5
1.08	0.566	tRNA ligase	2/21
1.03	0.639	Secretion	1/11

Interestingly, all three organisms show enrichments for tRNA ligases. The MoonProt database lists 15 tRNA ligases (mostly from eukaryotes) as having moonlighting functions. Note that the subject of the moonlighting-gene “local environment” continues in the “Discussion”, where it is suggested that the proximity of these genes to membrane and cell wall building genes is far from coincidental.

### Codon characteristics in moonlighting genes

**Figure 1:**
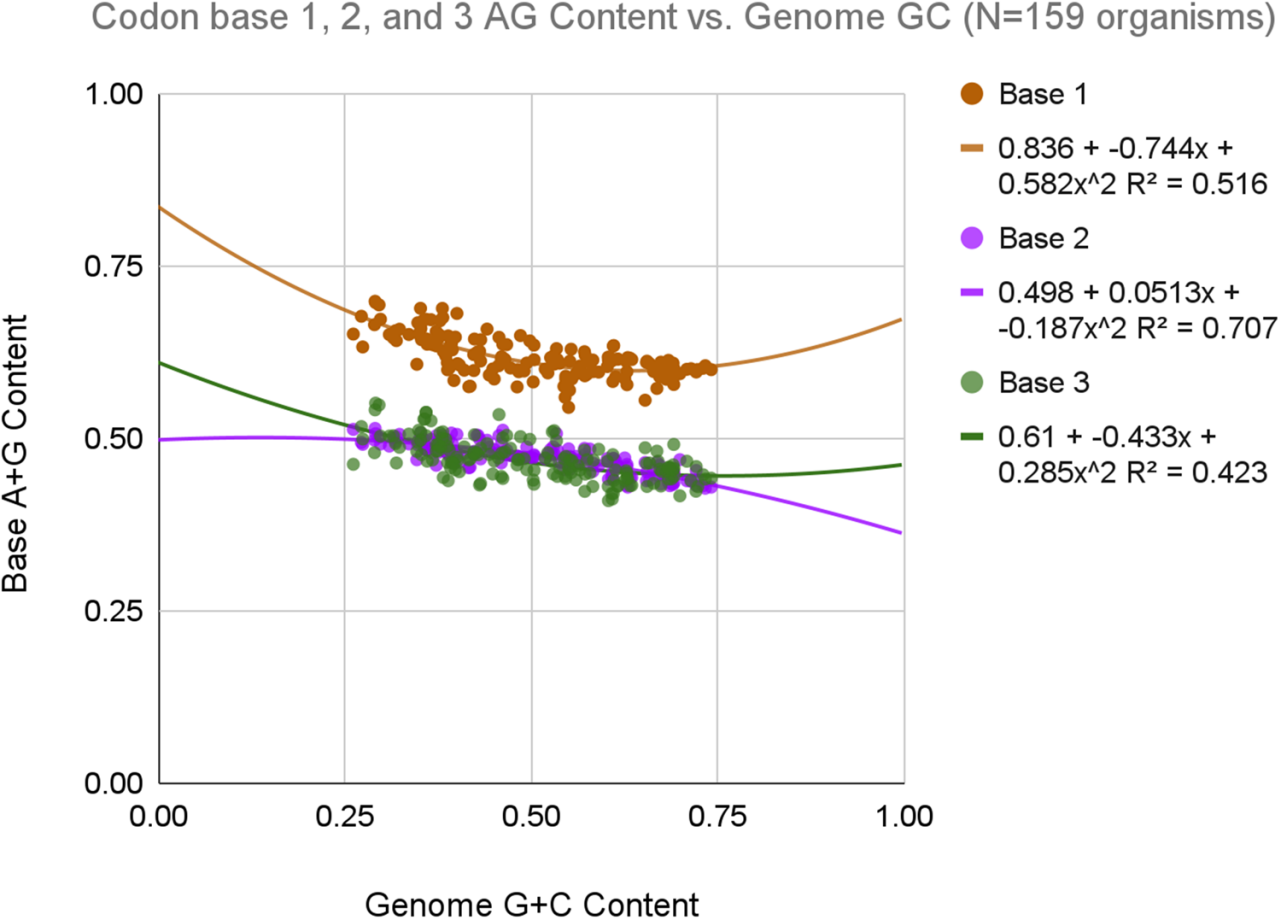
Purine content (A + G) of bases one, two, and three of codons for N = 159 bacterial genomes. Each dot represents a complete genome. See Supplement A for details.

In attempting to understand moonlighting genes, we began with what is arguably the single most basic and meaningful bioinformatic metric for studying any gene(s), which is the purine bias of base one of codons (hereinafter called R1). The significance of R1 (purine content, base 1) is that it is the single most reliable statistical indicator of open-reading-frame status. According to Ponce de Leon et al.^5^: “*It is the only sufficiently robust signal for assisting in gene searches and annotations within genome investigations.*” The reason is simple: Most genes, in most organisms, across all domains of life, have an average R1 value of 0.6 or more. That is, base 1 of codons in protein-coding genes is either adenine or guanine 60% of the time. While various explanations have been offered for this so-called “purine bias,” the simplest hypothesis is that the *DA-da-da-DA-da-da* cadence of this signal provides an easy way for the ribosome to detect and maintain frame alignment during translation. Figure 1 shows purine percent for each of the three bases of codons, versus CDS-genome G + C content, for N = 159 bacterial genomes. It can readily be seen that, for all organisms, the purine content of base one is significantly higher than for the other two codon bases.

**Figure 2:**
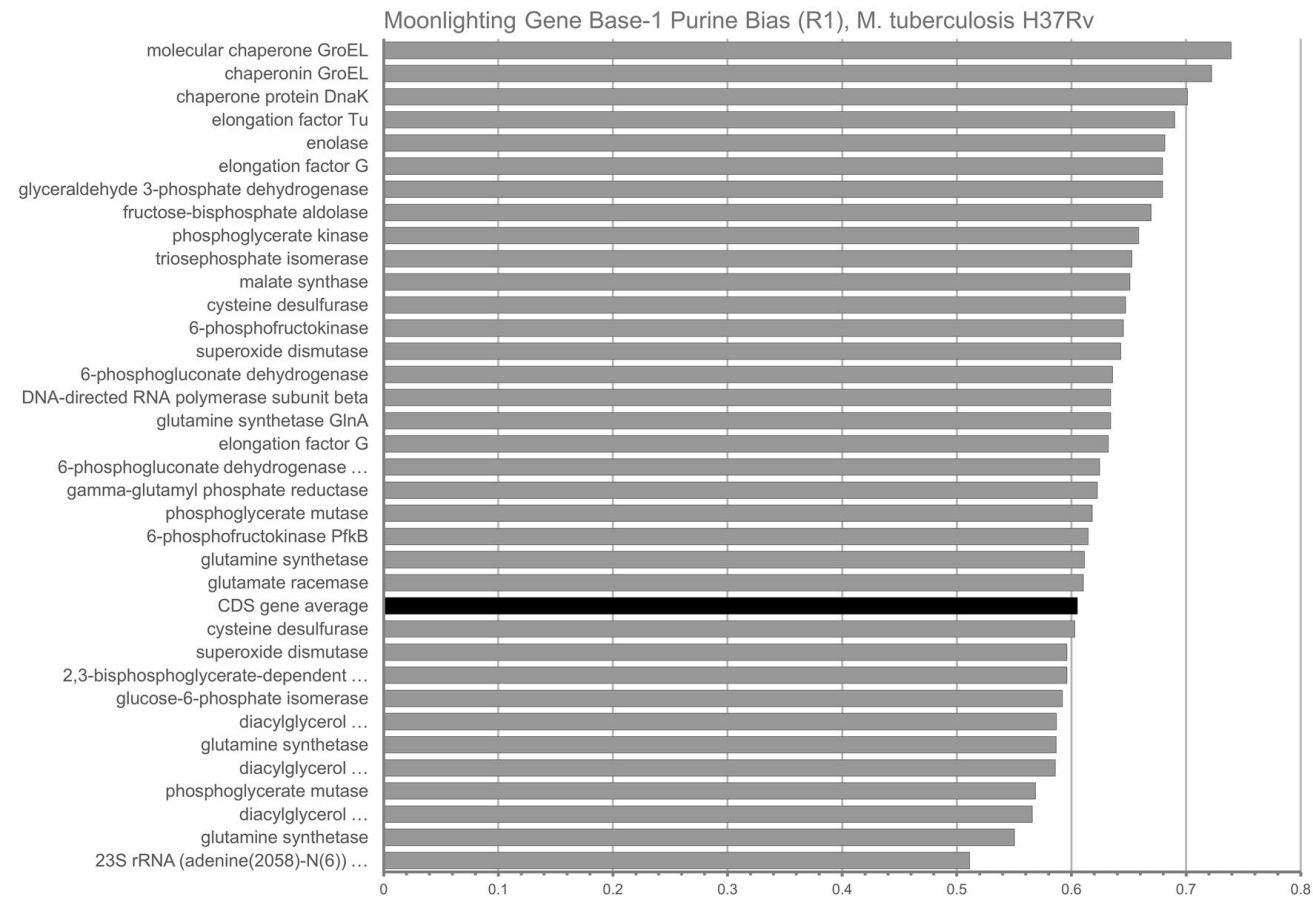
Purine content (R1) of base one of codons for N = 35 Moonlighting Genes in M. tuberculosis H37Rv. The genome-wide average of 0.60515 ± 0.05462 (for all 3906 CDS genes) is depicted in black.

In *M. tuberculosis*, the CDS-genome-wide mean value of R1 for all codons was found to be 0.60515 ± 0.05462. The R1 values for all moonlighting genes are graphed (Fig. 2). One can notice that a majority (24/35) of moonlighting genes show above-average R1 values.

**Figure 3:**
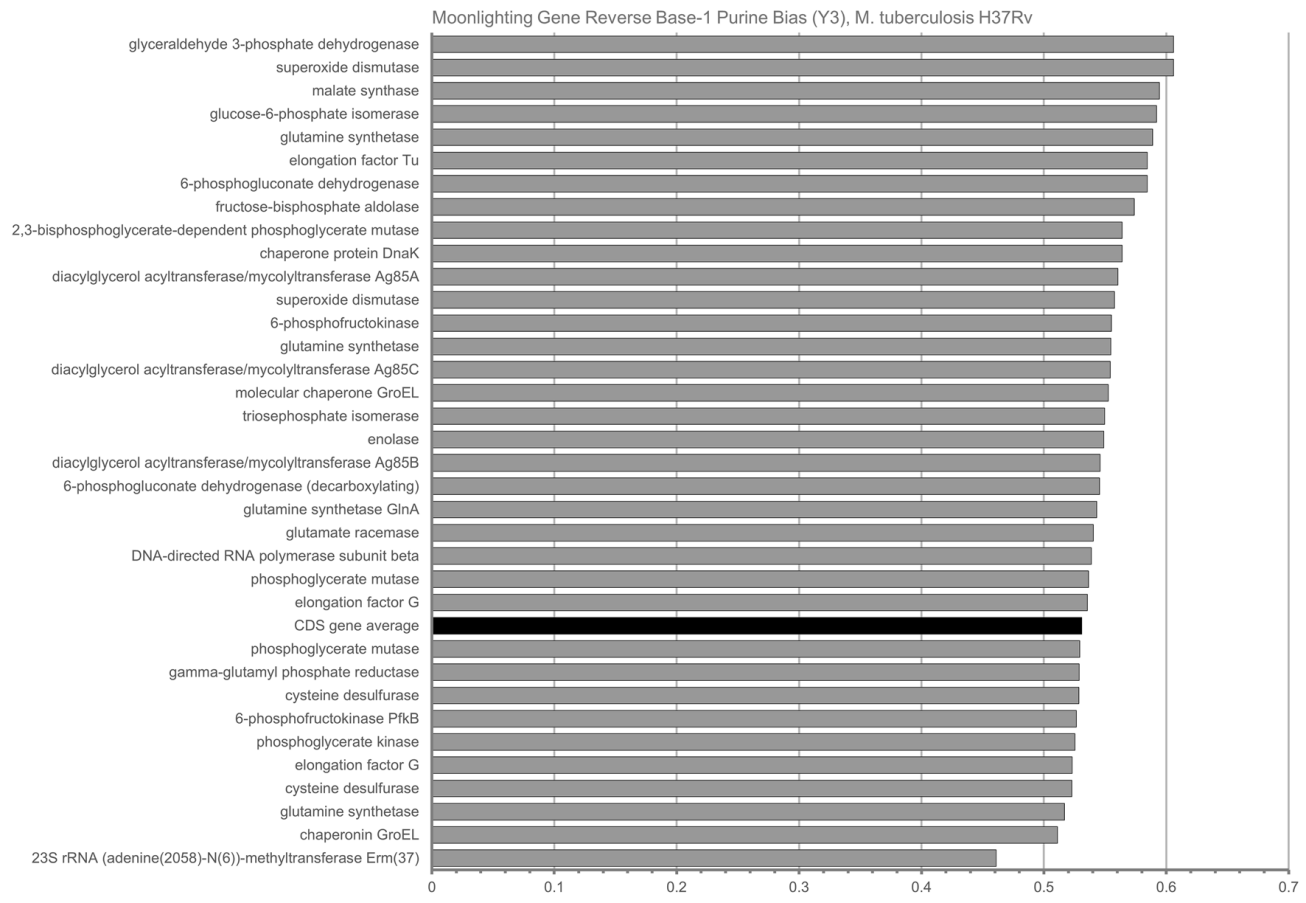
Reverse-complement purine content (R1) of base one of anti-codons for N = 35 Moonlighting Genes in M. tuberculosis H37Rv. The genome-wide average of 0.53089, 0.05213 ± 0.05213 (for all 3906 CDS genes) is depicted in black.

The unusually high R1 values for moonlighting genes caused us to wonder if R1 might also be high in the opposite direction, on the opposite strand of DNA. Figure 3 shows the base-one purine content for anti-codons of the same genes (that is, codons in the reverse-complement of the message strand). Somewhat surprisingly, 25/35 genes show above-average purine bias in reverse-complement codons.

### An enrichment assay based on codon metrics

The forward and reverse high R1 values of Figs. 2 and 3 suggest a strategy for obtaining enrichments of moonlighting genes: obtain R1_FORWARD_ and R1_REVERSE-COMPLEMENT_ for every gene in the CDS genome, add the two together, and sort all genes by that metric. Then take the top 20% of genes and see how many are moonlighting genes. When we did this, we found enrichments as shown in Table 6.

**Table 6:** Enrichment for moonlighting genes in *M. tuberculosis* H37Rv using R1-forward plus R1-rc (reverse complement) as a metric.

Fold	E	Function	Found/existing
4.67	0.000	PE-PGRS	56/60
2.24	0.000	Ribosomal protein	26/58
2.00	0.262	Peptidoglycan	2/5
**2.00**	**0.005**	**Moonlighting**	**14/35**
1.75	0.003	PPE family	22/63
1.67	0.488	Efflux	1/3
1.59	0.132	Esx	7/22
1.00	0.588	tRNA ligase	4/20

Significant values are in [bold].

All CDS genes were sorted by the metric and the top 20% (N = 781) analyzed. The expectation value (E) is the cumulative hypergeometric probability.

The top 20% of genes sorted by using the metric contain 14 of 35 moonlighting genes, for a 2.00-fold enrichment, at cumulative hypergeometric odds of 0.005. Next, we considered whether a metric summing R1-forward and R1-reverse would simply be equivalent to tallying the percent of codons that match the pattern RNY, where R is any purine, N is any base, and Y is any pyrimidine. Our analysis shows that the two metrics are not the same and they give slightly different results. The RNY metric is more effective (Table 7).

**Table 7:** Enrichment for Moonlighting Genes in M. tuberculosis H37Rv using RNY (purine-any base-pyrimidine) as a codon metric.

Fold	E	Function	Found/existing
4.67	0.000	PE-PGRS	56/60
3.00	0.057	Peptidoglycan	3/5
**2.29**	**0.000**	**Moonlighting**	**16/35**
1.83	0.001	PPE family	23/63
1.82	0.055	Esx	8/22
1.67	0.488	Efflux	1/3
1.21	0.258	Ribosomal protein	14/58
1.02	0.499	Transporter	17/83

Significant values are in [bold].

The enrichment for moonlighting genes in *M. tuberculosis* shows a value of 2.29-fold at an expectation of zero. This means moonlighting genes, more than other genes, contain codons matching the RNY pattern, a pattern that is inherently bidirectional (since the anticodon of RNY is also RNY). We naturally wondered if this result is limited to *M. tuberculosis*, or might apply generally, to other organisms. Thus, Tables 8 and 9 show the results for *E. coli* NCTC11775 and *Streptococcus pneumoniae* NCTC11032.

**Table 8:** Enrichment for moonlighting genes in *E. coli* NCTC11775 using RNY (purine-any base-pyrimidine) as a codon metric.

Fold	E	Function	Found/existing
**2.58**	**0.000**	**Moonlighting**	**16/31**
2.50	0.359	Anti-Sigma factors	1/2
2.46	0.000	Ribosomal protein	32/65
1.91	0.000	Membrane	32/84
1.22	0.013	Transporter	95/388
1.09	0.404	Efflux	15/69

Significant values are in [bold].

**Table 9:** Enrichment for moonlighting genes in *S. pneumoniae* NCTC11032 using RNY (purine-any base-pyrimidine) as a codon metric.

Fold	E	Function	Found/existing
**2.50**	**0.002**	**Moonlighting**	**10/20**
1.75	0.005	Ribosomal protein	20/57
1.50	0.322	Efflux	3/10
1.32	0.116	Permease	18/68
1.27	0.034	Transporter	48/189

Significant values are in [bold].

The RNY enrichment technique was effective in all three organisms. Also, notably, the gene functional categories were in good agreement across organisms; for example, ribosomal protein genes are generally enriched by this technique.

### Enhancement of enrichment

Two main questions arise in regard to the above results: (1) Could our enrichment technique be refined or improved in some way? (2) Why does the technique work at all? The presence of high purine bias in forward and backward directions suggests the potential for reverse transcription, and translation of antisense RNA, in these genes. We decided to pursue, as an Ansatz, the hypothesis that antisense open reading frames (asORFs) might exist in moonlighting genes. This led us to consider ways in which information running in two directions in the same gene could feasibly coexist. Our consideration was that the RY (purine/pyrimidine) axis might encode information differently than the SW (GC vs. AT) axis. Each axis is capable of encoding one bit’s worth of information. We wondered if degeneracy in bases one and three of codons might allow the “peaceful coexistence” of information on these axes, such that RY information going in one direction can effectively be superimposed on SW information going the other direction.

To test the above idea, a new metric was devised as follows:

1. For each gene, obtain the Shannon entropy of the RY signal in base one of codons. That is, find the average purine frequency (and pyrimidine frequency) for base one, and use it to calculate entropy in the standard way, as:
  $$entropy = f \times \left( \frac{1}{f} \right)$$
2. Do the same for base three.
3. Use the entropy values for base one and three to form a vector, [H_RY1_, H_RY3_].
4. Obtain the Shannon entropy of the SW signal (where ‘S’ means G or C and ‘W’ means A or T) in base one, and also in base three; and form a vector, [H_SW1_, H_SW3_].
5. Normalize the vectors so obtained.
6. Calculate their dot product. Use this as the basis of a metric.

The dot product of two vectors measures how much the vectors differ, directionally, because the dot product of normalized vectors is the cosine of the angle between them. A large difference is expected for the RY and SW vectors in the case of moonlighting genes. We expect a large angle and a small cosine, hence the score value for genes is computed according to *1−cosine*. Enrichment values for *M. tuberculosis* are shown in Table 10, where genes are ranked by this new metric.

**Table 10:** Enrichment for moonlighting genes in *M. tuberculosis* H37Rv using a metric based on the dot product of RY and SW entropy vectors (see text for “Discussion”).

Fold	E	Function	Found/existing
4.17	0.000	Mycolate synthesis	10/12
2.59	0.000	Ribosomal protein	30/58
2.58	0.000	PE-PGRS	31/60
2.43	0.000	Moonlighting	17/35
1.82	0.055	Esx	8/22
1.78	0.010	Permease	16/45
1.67	0.488	Release factor	1/3
1.67	0.488	Efflux	1/3
1.11	0.433	Fatty-acid synthesis	8/36
1.10	0.325	Transmembrane	27/123
1.07	0.343	Membrane	41/192
1.03	0.499	PPE family	13/63

Enrichment is based on the top 20% of genes.

Results shown in Table 10 provide several new additional functional categories. The top four—including moonlighting genes—have fold-enrichments exceeding 2.0 and expectation values of zero. Further enrichment occurs when the RNY metric is combined with the entropy-dot-product-based metric. By simply summing the two metrics together (to produce a new metric), we were able to find 20 out of 35 moonlighting genes in *Mycobacterium tuberculosis* H37Rv, for a fold-enrichment of 2.86 at expectation zero. This same metric yields a 2.58-fold enrichment (E = 0) for moonlighting genes in *E. coli,* and a 2.25-fold enrichment in *S. pneumoniae* (at E = 0.009).

### Antisense translation products: theoretical considerations

**Figure 4:**
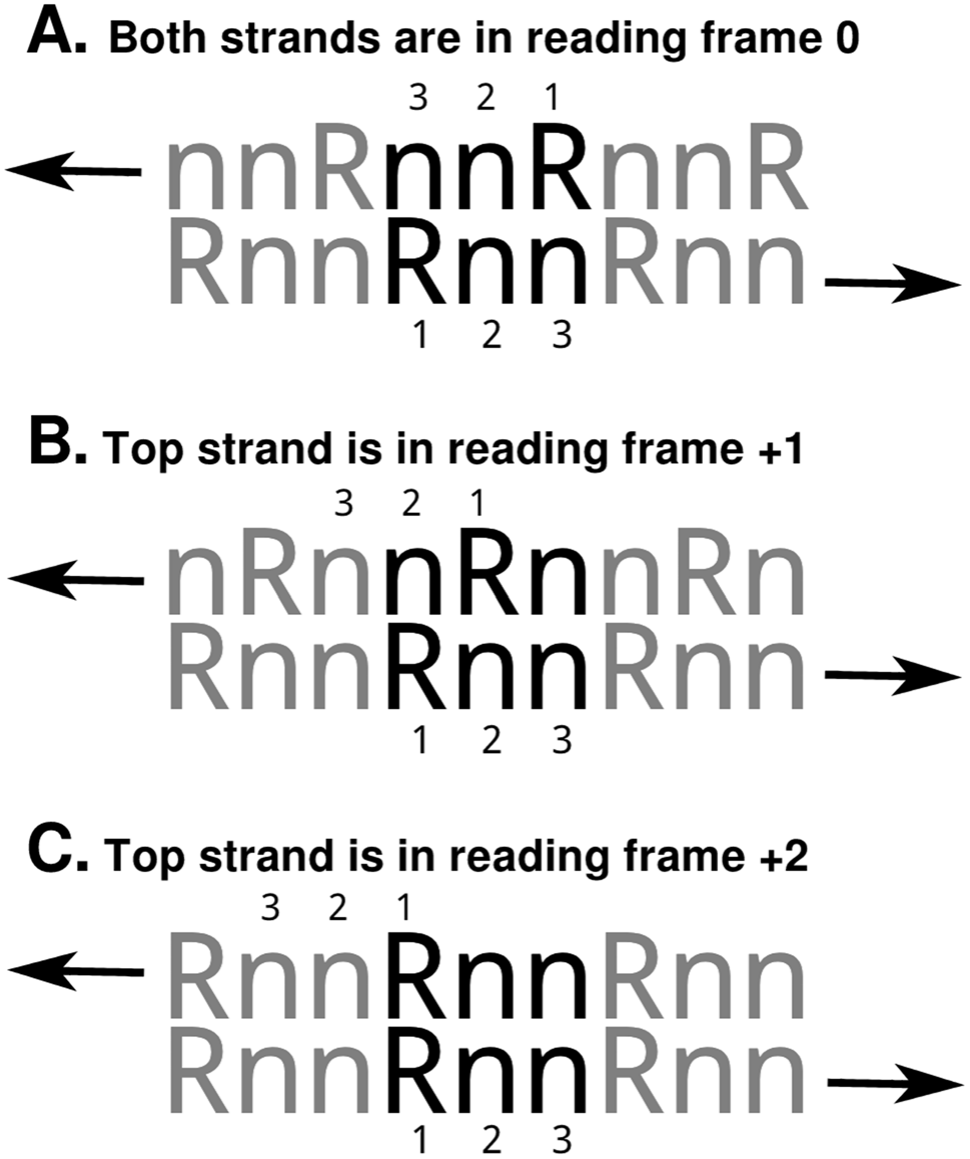
Possible reading frame alignments in forward and reverse strands. Arrows designate the reading direction; the number “1 2 3” indicate codon base positions. ‘R’ means any purine; ‘n’ means any base. The ‘Rnn’ pattern is representative of ~ 60% of codons in any organism, across all domains of life. The top configuration (**A**) shows both strands in reading frame zero. In this configuration, base 2 of codons and anticodons align. In the middle configuration (**B**), the top strand is in reading frame + 1 (relative to the beginning of that strand) while the bottom strand is in reading frame zero. In this alignment, base 3 of codons occur opposite base 3 of anticodons. But notice that base 2 occurs opposite a purine, which means base 2 will be a pyrimidine. In the bottom configuration (**C**), the top strand is in reading frame + 2 (or equivalently, − 1), which means base one of codons will occur opposite base 1 of anticodons. This is not a feasible alignment if both codon and anticodon have a purine in the first-base position. See text for further “Discussion”.

Based on the above results, which are consistent with our Ansatz (which says that antisense ORFs might exist in moonlighting genes), we decided to look for open reading frames in the reverse complements of moonlighting genes in our three model organisms. Until now, we have assumed naively, based on purine bias in reading frame zero, that antisense products will exist in frame zero on the complement strand. (We consider that there are three possible reading frames: zero, + 1, and + 2. These frames can exist on either strand, relative to the 5’ terminus of the strand.) But is this really a reasonable expectation? On purely theoretical grounds, we consider that there are three possible reading frames in the reverse direction, with different implications for overlap of codon information. Forward and reverse reading frames can overlap in the following ways (Fig. 4):

In Fig. 4, complementary strands of DNA are shown adjacent each other, with codon bases numbered 1–2–3 on the bottom strand (which reads left to right, in this depiction) and anticodon bases numbered 3–2–1 on the top strand (which reads right to left). The symbol ‘R’ represents a purine; ‘n’ is any base. Arrows represent reading directions. At the top of the diagram, in the section labeled ‘A’, strands are oriented in “2 over 2” fashion: base 2 of the codon is opposite base 2 of its anticodon. This is the orientation that occurs if the translation reading frame is zero (the default) for each strand. The middle portion of the diagram, labeled ‘B’, shows codon/anticodon orientation when the top strand is in reading frame + 1. In this case, base 3 of one codon overlaps base 3 of the other; a so-called “3-over-3” orientation. The lowermost pair of strands (labeled ‘C’ in the diagram) shows the situation where the bottom strand is (as usual) in reading frame zero but the top strand is in reading frame + 2 (or, equivalently, − 1). This puts base one of the codon opposite base one of the anticodon (“1 over 1” configuration).

Clearly, in a gene that has overlapping ORFs, the “1 over 1” configuration is not feasible if each ORF has high purine bias, because a purine can never occur opposite to another purine in DNA. Therefore, we expect a + 2 antisense reading frame to be a rare occurrence. It could exist, but only if one ORF has low purine bias and the other strand has high purine bias; or if both strands have 50% purine bias.

The top configuration (2 over 2), where both strands are in reading frame zero, is at least tenable, since high purine content in base one of either strand can be offset by correspondingly high pyrimidine content in base three. This is feasible since base three is mostly degenerate; the requirement for a pyrimidine in base three comes at little cost. Nevertheless, “2 over 2” means that if base two is predominantly purines in one ORF, it *must* be predominantly pyrimidines in the other ORF. Thus, if the bottom strand encodes a hydrophilic polypeptide, the top strand will likely encode a hydrophobic one—a membrane protein.

The middle configuration (3 over 3), which has the top strand in reading frame + 1, will accommodate high purine bias in both strands simultaneously *iff* base two of each codon is a pyrimidine. Crucially, this means *each* ORF must encode a polypeptide with high non-polar amino acid content. (The genetic code is arranged in such a way that a pyrimidine in base two is very likely to mean a non-polar amino acid). This, in turn, means *both* translation products are likely to be membrane proteins.

To summarize, we expect that if an antisense ORF exists in a gene, it will only rarely be in reading frame + 2; it may be in frame zero; and it is reasonably likely to be in the + 1 frame, particularly if membrane proteins are the translation products.

### Theory validation

The following question arises from the above logical deductions: How can we look for antisense ORFs? Where will they begin? The trivial answer is, they begin with a start codon; and they meet the requirement of an ORF, which is to say:

1. The start codon should be preceded by something resembling a Shine Dalgarno sequence.
2. The start codon should be ATG or perhaps GTG.
3. The start codon should be followed by some (suitably large) number of codons having significant purine bias in base one.
4. And the sequence of codons should end in a stop codon (TAA, TAG, or TGA).

A search for such structures can be performed by obtaining the reverse complement of a gene and matching it against a regular expression, such as:

$$/.\left\{ {{14}} \right\}\left( {\left[ {{\text{GA}}} \right]{\text{TG}}} \right)\left( {...} \right)\left\{ {{1},} \right\}?\left( {? = \left( {{\text{TAA}}\left| {{\text{TAG}}} \right|{\text{TGA}}} \right)} \right).../{\text{g}}$$

This expression allows a search for any 14 bases, followed by ATG or GTG, followed by 1 or more triplets that do not include a stop codon, followed by three bases. (The ‘g’ at the end simply means to search globally and report multiple results). The 14-base leader can be checked for Shine Dalgarno motifs (or a proxy measure, such as purine percentage) so as to filter low-quality hits. In order to obtain the translatable portion of a hit, the first 14 bases can simply be removed (or ignored). Hits might contain any number of codons; it is up to the user to set a suitable minimum limit. While the above regular expression produces good results, we find, in practice, that better results are obtained using:

$$/.\left\{ {{12}} \right\}\left( {..\left[ {{\text{ATG}}} \right]{\text{TGA}}..} \right)\left( {...} \right)\left\{ {{1},} \right\}?\left( {? = \left( {{\text{TAA}}\left| {{\text{TAG}}} \right|{\text{TGA}}} \right)} \right).../{\text{g}}$$

This expression searches for the combination start/stop motifs ATGA, TTGA, and GTGA, which are common in leaderless genes of all three of our model organisms (unpublished data). The first three bases of the motif constitute a start codon; the last three bases constitute the “opal” stop codon, TGA. When this motif occurs in leaderless genes, translation halts at the TGA, then begins again after a – 1 frameshift.

When we looked for putative antisense ORFs in the moonlighting genes of our three model organisms using the start/stop-motif regex, we found hits in almost every gene (see Table 11). Most of the hits (90/142 = 63.4%) were in the + 1 reading frame, as predicted.

**Table 11:** Summary: putative antisense ORFs in moonlighting genes of *M. tuberculosis, E. coli*, and *S. pneumoniae*.

Stat	*M. tuberculosis*	*E. coli*	Strep
Moonlighting genes containing hits	34/35	27/31	20/20
Total regex hits	56	48	38
Average ORF length (bases)	404	394	357
Reading frame 0	7	12	9
Reading frame + 1	41	29	20
Reading frame + 2	8	7	9
Leader purine %	0.512	0.523	0.528
R1 (ave. purine content, base 1)	0.527	0.526	0.519
Y2 (Ave. pyrimidine content, base 2)	0.588	0.605	0.554

Reverse-complemented genes were searched using the regular expression /.{14}([GA]TG)(…){90,}?(? = (TAA|TAG|TGA))…/g; see text for detail.

While values of R1 (average purine content, base 1 of codons) are seemingly quite low in the hits, this is largely due to the abnormally low R1 values of the reading-frame + 2 hits, which drag the averages down. When we look at R1 values by reading frame (Table 12), we see that R1 values for reading frames zero and + 1 are reasonably high. Most likely, the reading-frame + 2 hits can be considered false positives. Some false positives also likely occur in reading-frames zero and + 1.

**Table 12:** Summary: average R1 (purine bias, base 1) of putative antisense ORFs in moonlighting genes of *M. tuberculosis, E. coli*, and *S. pneumoniae*.

Organism	Frame 0	Frame + 1	Frame + 2
*M. tuberculosis*	0.5524	0.5463	0.4042
*E. coli*	0.5861	0.5310	0.4000
*S. pneumoniae*	0.6259	0.5317	0.3852

An analysis of Y2 (pyrimidine content, base 2 of codons) shows that Y2 is significantly higher in reading-frame + 1 hits than in other reading frames (Table 13), in agreement with our prediction (see previous section) and suggests that any putative antisense ORFs that exist in frame + 1 likely encode membrane proteins. Overall, the results in Tables 11, 12, and 13 are in strong agreement with the theoretical predictions of the previous section.

**Table 13:** Summary: average Y2 (pyrimidine content, base 2) of putative antisense ORFs in moonlighting genes of *M. tuberculosis, E. coli*, and *S. pneumoniae*.

Organism	Frame 0	Frame + 1	Frame + 2
*M. tuberculosis*	0.4048	0.6309	0.5250
*E. coli*	0.4472	0.7046	0.4619
*S. pneumoniae*	0.4593	0.6700	0.3926

### Putative antisense ORFs contain transmembrane domains

We attempted to gain further insight into whether the translation products of putative antisense ORFs that occur in moonlighting genes might, in fact, encode membrane proteins. To do this, we searched for antisense-direction hits using regular expressions based on:

$$/.\left\{ {{14}} \right\}\left( {{\text{ATG}}} \right)\left( {...} \right)\left\{ {{1},} \right\}?\left( {? = \left( {{\text{TAA}}\left| {{\text{TAG}}} \right|{\text{TGA}}} \right)} \right).../{\text{g}}$$

The above expression uses ATG as the presumptive start codon. However, we also used expressions containing start codons TTG, GTG, and CTG, as well as alternate start codons ATA, ATT, ATC, and TAC, based on an examination of the start codons in the annotated genes of our three model organisms. The three model organisms use all of these start codons (according to the annotated RefSeq genomes). We conducted separate regex searches using each start codon. Once putative ORFs were located, we translated the ORFs in silico and submitted the resulting polypeptide sequences to the Consensus Constrained TOPology (CCTOP) server app at http://cctop.ttk.hu/. CCTOP is a web application that takes a consensus-based approach to predicting transmembrane topologies. Using 10 different topology prediction methods, CCTOP incorporates previously determined structural and topology information into a probabilistic Hidden Markov Model. Its reliability (in terms of reduced false positives and false negatives) has been demonstrated to be better overall than HMMTOP, Phobius, or any other single transmembrane prediction technology used alone. Details can be found at the CCTOP website or in Dobson et al.^6^.

CCTOP predicted transmembrane domains in seven antisense ORFs from moonlighting genes of *M. tuberculosis* (Table 14). Some of the ORFs were overlapping. For example, glutamine synthetase subunit A1 (*glnA1*) was found to contain a putative antisense ORF with start codons ATG and TTG at offsets 613 and 625. Likewise, *rpoB* was found to contain an antisense ORF with three closely spaced start codons.

**Table 14:** Antisense ORFs containing predicted transmembrane domains, moonlighting genes of *M. tuberculosis*.

Gene	Name	Offset	Length (bases)	Start codon	RF	AA sequence
dnaK	Chaperone protein DnaK	270	804	TAC	0	YLRTTLGLTLFFDELLRLVDQCLGLITNIGLLATLAILLGVRFGVLDHAVNVFLGQARAFLDSDRVLLAGALVLGGDVHNAVGVDVESDLDLRNPPRRRRDAGQLEGPEQLVVRGDLTLPLIDLDLHRRLVVVGGGESLRPLGGDRGVALDEPGHHPALGLDTQAQRGNIKQQNVFHLALEDAGLQSGSHRDNLIGVDALVGFLAAGEFLDQIGHRGHPGRTTHEHNVIDLRHRNAGVSDHRLERLASAVQQVLSDPLELRAGQLLV
dnaK	Chaperone protein DnaK	412	222	ATG	1	MRSMSSLDRPEPSWIRIVFSLPVPLSLAVTCTMPLASMSKVTSICGIPRGAGGMPVSSKDPSNLLCAAISRSP
fba	Fructose-bisphosphate aldolase	433	291	TTG	1	LPCSPAPSASMVFSKSSGLVYSFSLISFATPSSSPPTTPISISRMILAAAAALSSSWAMARFSSIGTAEPSHMCDWNKGLPPLLTRCAEIASKGRT
glnA1	Glutamine synthetase	613	138	ATG	1	MPDPLSPNSGLGMNVTVLPFCQAVFLMMYLYNCMSSAACSSELNW
glnA1	Glutamine synthetase	625	126	TTG	1	LSPNSGLGMNVTVLPFCQAVFLMMYLYNCMSSAACSSELNW
gnd2	6-Phosphogluconate dehydrogenase (decarboxylating)	736	123	TTG	1	LPPSITMSPASSVLASSSITAVVMFPAGTITQTTRGAESR
iscS	Cysteine desulfurase	1075	93	CTG	1	LVCSDDALPMVRCTAAIASMAAGCIGVVAA
proA	Gamma-glutamyl phosphate reductase	139	390	GTG	1	VNAVDAFTITAAASICSVKRWAASRLVVTIASVCPVPYSLIWAMAASTPSTTATAMSSDRYSRRRSASSGSRCTVTPACCRAASNRGNAVSAIAASTSSVSAALQTLGRRVLEFSKIRSATSRSAAWCT
rpoB	DNA-directed RNA polymerase subunit beta	2182	303	TTG	1	LWVNPDSGLFWSMNWLSWLVPKNSLIAATTGRMLINVCGVIASTSWVVIRSRTTRSIRDMPTRIWFWISSPTVRRRRLPKWSMSSVSTGTSTPPGTVIVV
rpoB	DNA-directed RNA polymerase subunit beta	2218	267	ATG	1	MNWLSWLVPKNSLIAATTGRMLINVCGVIASTSWVVIRSRTTRSIRDMPTRIWFWISSPTVRRRRLPKWSMSSVSTGTSTPPGTVIVV
rpoB	DNA-directed RNA polymerase subunit beta	2254	231	TTG	1	LIAATTGRMLINVCGVIASTSWVVIRSRTTRSIRDMPTRIWFWISSPTVRRRRLPKWSMSSVSTGTSTPPGTVIVV

Offsets are in the antisense direction. Length of putative ORF is in bases. RF, reading frame (antisense direction).

Fifteen moonlighting genes of *E. coli* yielded 49 asORFs with predicted transmembrane domains (Table 15). The genes were: *aceB, dnaK, eno, fusA, gapA, glcB, gpmB, gpmM, murI, pfkA, pfkB, pgi, rpoB, sodC*, and *tuf*. Again, as with *Mycobacterium tuberculosis* H37Rv, many of the hits overlap: for example, in *fusA*, start codons for what appears to be a single antisense ORF exist at offsets 682, 697, and 709. Notably, *rpoB* contains at least four closely spaced “start codons” inside a putative antisense ORF that spans 2892 bases in total length.

**Table 15:** Antisense ORFs containing predicted transmembrane domains, moonlighting genes of *E. coli*.

Gene	Name	Offset	Length (bases)	Start codon	RF	AA sequence
aceB	Malate synthase A	261	408	ATC	0	IFHQAINRHTAIARDPRFDVLHSHANVGAHTFFGAFTITRCQQLIGSNRRVLFAHHLKLVFAGAENIVEYRHCRISKARVSDPCAIVTVIGFQRFIRFYFVEHLVIALFIFAWNKRRHAAHRKSTAFMAGFNQQA
aceB	Malate synthase A	276	393	ATA	0	INRHTAIARDPRFDVLHSHANVGAHTFFGAFTITRCQQLIGSNRRVLFAHHLKLVFAGAENIVEYRHCRISKARVSDPCAIVTVIGFQRFIRFYFVEHLVIALFIFAWNKRRHAAHRKSTAFMAGFNQQA
aceB	Malate synthase A	294	375	ATT	0	IARDPRFDVLHSHANVGAHTFFGAFTITRCQQLIGSNRRVLFAHHLKLVFAGAENIVEYRHCRISKARVSDPCAIVTVIGFQRFIRFYFVEHLVIALFIFAWNKRRHAAHRKSTAFMAGFNQQA
aceB	Malate synthase A	321	348	CTG	0	LHSHANVGAHTFFGAFTITRCQQLIGSNRRVLFAHHLKLVFAGAENIVEYRHCRISKARVSDPCAIVTVIGFQRFIRFYFVEHLVIALFIFAWNKRRHAAHRKSTAFMAGFNQQA
aceB	Malate synthase A	390	279	CTG	0	LIGSNRRVLFAHHLKLVFAGAENIVEYRHCRISKARVSDPCAIVTVIGFQRFIRFYFVEHLVIALFIFAWNKRRHAAHRKSTAFMAGFNQQA
aceB	Malate synthase A	468	201	TAC	0	YRHCRISKARVSDPCAIVTVIGFQRFIRFYFVEHLVIALFIFAWNKRRHAAHRKSTAFMAGFNQQA
aceB	Malate synthase A	498	171	GTG	0	VSDPCAIVTVIGFQRFIRFYFVEHLVIALFIFAWNKRRHAAHRKSTAFMAGFNQQA
aceB	Malate synthase A	1171	276	ATG	1	MVPLTASRRLICPSITLFQSGASESSKSAIKTFTLALSALITILRSTGPVISTRRSSKSEGIPRIFQSASRMEAVSEIKSGNIPLSISCCC
dnaK	Molecular chaperone DnaK	604	183	ATG	1	MVTADWLSSAVENTWLCLVGIVVFFAISVVITPPMVSIPRDSGVTSSSSTSFTSPVRTPP
dnaK	Molecular chaperone DnaK	622	165	TTG	1	LSSAVENTWLCLVGIVVFFAISVVITPPMVSIPRDSGVTSSSSTSFTSPVRTPP
dnaK	Molecular chaperone DnaK	649	138	CTG	1	LCLVGIVVFFAISVVITPPMVSIPRDSGVTSSSSTSFTSPVRTPP
dnaK	Molecular chaperone DnaK	916	174	ATA	1	ISDTDRPASCSATFSGSMERFTRSSTRLSSFARVTLMFMCFGPVASAVMYGRLTSVC
dnaK	Molecular chaperone DnaK	967	123	ATG	1	MERFTRSSTRLSSFARVTLMFMCFGPVASAVMYGRLTSVC
eno	Phosphopyruvate hydratase	787	255	ATC	1	IMNSWISTLLSACSPPLMMFIIGTGIEYLPGVPFSSAMCSYSGIPLAAAAALALARDTARIAFAPNLDLFSVPSRSIMILSMPA
eno	Phosphopyruvate hydratase	790	252	ATG	1	MNSWISTLLSACSPPLMMFIIGTGIEYLPGVPFSSAMCSYSGIPLAAAAALALARDTARIAFAPNLDLFSVPSRSIMILSMPA
eno	Phosphopyruvate hydratase	838	204	ATG	1	MMFIIGTGIEYLPGVPFSSAMCSYSGIPLAAAAALALARDTARIAFAPNLDLFSVPSRSIMILSMPA
fusA	Elongation factor G	682	342	TTG	1	LNSRFIRSTMMSRCSSPIPAMMVWLDSSSVHTRKDGSSLARRPRARPIFSWSALVFGSTAMEITGSGNSIRSRMIGASGSHRVSPVVTSFRPIAAAMSPARTSLISSRLLACI
fusA	Elongation factor G	697	327	ATA	1	IRSTMMSRCSSPIPAMMVWLDSSSVHTRKDGSSLARRPRARPIFSWSALVFGSTAMEITGSGNSIRSRMIGASGSHRVSPVVTSFRPIAAAMSPARTSLISSRLLACI
fusA	Elongation factor G	709	315	ATG	1	MMSRCSSPIPAMMVWLDSSSVHTRKDGSSLARRPRARPIFSWSALVFGSTAMEITGSGNSIRSRMIGASGSHRVSPVVTSFRPIAAAMSPARTSLISSRLLACI
gapA	Glyceraldehyde-3-phosphate dehydrogenase	490	318	ATG	1	MMPKLSLITLASGARQLVVQEALETMSWPAYLSKLAPLTNIGVLSLDGPVITTFFAPAVMCLRAVSSVRNRPVASATTSTPTSSHFRLAGSRSAVTRIFLPLTIR
gapA	Glyceraldehyde-3-phosphate dehydrogenase	493	315	ATG	1	MPKLSLITLASGARQLVVQEALETMSWPAYLSKLAPLTNIGVLSLDGPVITTFFAPAVMCLRAVSSVRNRPVASATTSTPTSSHFRLAGSRSAVTRIFLPLTIR
glcB	Malate synthase G	328	204	ATC	1	IPCSTQRTTYPRIPCTLLSSSCWISCADQLAFSATGIVSRSSSSGSNSALNSVWAMLACTLCTLVWW
glcB	Malate synthase G	436	96	ATA	1	IVSRSSSSGSNSALNSVWAMLACTLCTLVWW
gpmB	2,3-Diphosphoglycerate-dependent phosphoglycerate mutase GpmB	193	396	ATA	1	IPWLTSSGRLPCGKSRQDSSAALTRSLSSCIDSPSGIRPSTVPLTSCRRQFSSSSVSESIFLVSSTPIFNSRRRESKMMSQPQAWAMISAVRRVRPKSLLMICVMPSSLARVATCIACCSPLAVSGLSDWP
gpmB	2,3-Diphosphoglycerate-dependent phosphoglycerate mutase GpmB	271	318	CTG	1	LSSCIDSPSGIRPSTVPLTSCRRQFSSSSVSESIFLVSSTPIFNSRRRESKMMSQPQAWAMISAVRRVRPKSLLMICVMPSSLARVATCIACCSPLAVSGLSDWP
gpmB	2,3-Diphosphoglycerate-dependent phosphoglycerate mutase GpmB	283	306	ATT	1	IDSPSGIRPSTVPLTSCRRQFSSSSVSESIFLVSSTPIFNSRRRESKMMSQPQAWAMISAVRRVRPKSLLMICVMPSSLARVATCIACCSPLAVSGLSDWP
gpmB	2,3-Diphosphoglycerate-dependent phosphoglycerate mutase GpmB	316	273	GTG	1	VPLTSCRRQFSSSSVSESIFLVSSTPIFNSRRRESKMMSQPQAWAMISAVRRVRPKSLLMICVMPSSLARVATCIACCSPLAVSGLSDWP
gpmB	2,3-Diphosphoglycerate-dependent phosphoglycerate mutase GpmB	358	231	GTG	1	VSESIFLVSSTPIFNSRRRESKMMSQPQAWAMISAVRRVRPKSLLMICVMPSSLARVATCIACCSPLAVSGLSDWP
gpmB	2,3-Diphosphoglycerate-dependent phosphoglycerate mutase GpmB	427	162	ATG	1	MSQPQAWAMISAVRRVRPKSLLMICVMPSSLARVATCIACCSPLAVSGLSDWP
gpmM	2,3-Bisphosphoglycerate-independent phosphoglycerate mutase	1168	144	CTG	1	LCTPPAESRPIMCTALPAFFALSTAPVNTGLAKKARSLISTSRRVRS
murI	Glutamate racemase	170	234	CTG	2	LRQNPPAGFPLAAPVTVLLVVEGNGYTPVQRYLAALSFLTTGVGYVLAHPEKHLRHAASLQPTQPSLPSPAFLSGIH
pfkA	6-Phosphofructokinase	211	237	ATG	1	MWPSTVARVSRPVSFSMKCASSSTSHICSVIATIACFLPFAIPALISFTRSSRLNSTSGTTTNSQPPAMAAANVRSPQ
pfkA	6-Phosphofructokinase	259	189	ATG	1	MKCASSSTSHICSVIATIACFLPFAIPALISFTRSSRLNSTSGTTTNSQPPAMAAANVRSPQ
pfkA	6-Phosphofructokinase	289	159	ATA	1	ICSVIATIACFLPFAIPALISFTRSSRLNSTSGTTTNSQPPAMAAANVRSPQ
pfkB	6-Phosphofructokinase II	64	150	TTG	1	LSVAALPAATPKRTISSREAFSASFSVIAPTMLSPAPTVLWLFTGGGTT
pgi	Glucose-6-phosphate isomerase	18	810	ATA	0	IAVNQTIGRAIVAADFFIIFQLWQNTVRQLFTQLHAPLVEGEDVQDHALSKDFVLIQRNQRTQAERSDFTQQDGVGRAVTFEHFERHHVVKRCRIFTLIAIFLLNHFAGFTKRQRFGLSKEVRQQFLVMIRERVMGDSRSDEIARYHFGSLVDQLIERVLTVRARFTPDNRASLVIHNLTVTVNILTVGFHIALLEVRRETVHILVIRQNRFSFRAKEIVVPDANQRQQYRQVFLGRRGGEMLVHRVCARKQFNEVIKADGENNRQANR
pgi	Glucose-6-phosphate isomerase	399	429	GTG	0	VMIRERVMGDSRSDEIARYHFGSLVDQLIERVLTVRARFTPDNRASLVIHNLTVTVNILTVGFHIALLEVRRETVHILVIRQNRFSFRAKEIVVPDANQRQQYRQVFLGRRGGEMLVHRVCARKQFNEVIKADGENNRQANR
pgi	Glucose-6-phosphate isomerase	402	426	ATG	0	MIRERVMGDSRSDEIARYHFGSLVDQLIERVLTVRARFTPDNRASLVIHNLTVTVNILTVGFHIALLEVRRETVHILVIRQNRFSFRAKEIVVPDANQRQQYRQVFLGRRGGEMLVHRVCARKQFNEVIKADGENNRQANR
pgi	glucose-6-phosphate isomerase	453	375	TAC	0	YHFGSLVDQLIERVLTVRARFTPDNRASLVIHNLTVTVNILTVGFHIALLEVRRETVHILVIRQNRFSFRAKEIVVPDANQRQQYRQVFLGRRGGEMLVHRVCARKQFNEVIKADGENNRQANR
rpoB	DNA-directed RNA polymerase subunit beta	87	2892	CTG	0	LMVAVHDVFIHLSTAVHVIRLNGEHFLQGVCCAICFQRPHFHLPETLTTELCLTTQRLLSNQAVRTGGTRVHLVVDQVVQFQHVHVTNGYRTLELFTSATVVQADLTRSRQVAKFQQLFNFCFFRTVEDRRCDWHTFAQVFSQTHNFFIAEGTQVNFLTNISAQIVRTLDEFAQFRDFLLLFQHGVDLVADTFRSHTQVGFEDLTNVHTRRYAQRVQYDVYRSTVFIVRHIFDRVDLRNYTLVTMTACHLVTRLDTAFNRQIYLNNLQHARCQIVALGDFAAFRFEFLLELVFQFVILLSQLFQLILFLFVGQAQLQPAIARQFVEFLSFNATGYQHSTDTAEQTRFEDLQFFRQVFLRLFELHFFDFQRTFVFFYAIASKDLNIDNRTGYTVWYAQRRVFNVRRFLTEDRTQQFFFWGQLSFTFRRYLTNQNVATGHFRTNVNDTGFIQFGESSFTHVRDVSGDLFRPQLGITGHTRQFLNMDGGETVFLNNTLGYEDGVFEVVTIPRHERYAHVLTKRQFTEIGGRTVCQHVAAFYRFTQRHTRHLVDTGVLVRTGVLGQVVDVDTCFTRIHLVFVNFDNDTGSIHVLNDTTTFSNRSYTGVNGNSTFHTRTNQRLISTQSRNGLTLHVRTHQCTVGVIVFQERDQGRTDGYHLLGGYVHVVNLVAAEQAGFAFATASYQVFYEVAFFIQVGVRLGDNVVAFFDSRQIVNFVSYNTVGHFTIRSLKEAVFVSLCVHGQGVDQTDVRTFRGFDWTYATVVSRVYVSNFEACTFTGQTAWAECRDTTFVRNLRQRVVLVHKLRQLAGTEELFHCCGNRLGVDHILRHQGIQIAQRQTLFHRTLYTYQANAELVFRHFANRTDTTVAEVVDIINFAFTVTDIDELFHNINDVVFAQDTGTFDFFAQQRTVELHTTNRRQVIAVFGEEQVLEQAFSSFTSRRLARAHHTVDFYQCAQTVVSWVDT
rpoB	DNA-directed RNA polymerase subunit beta	90	2889	ATG	0	MVAVHDVFIHLSTAVHVIRLNGEHFLQGVCCAICFQRPHFHLPETLTTELCLTTQRLLSNQAVRTGGTRVHLVVDQVVQFQHVHVTNGYRTLELFTSATVVQADLTRSRQVAKFQQLFNFCFFRTVEDRRCDWHTFAQVFSQTHNFFIAEGTQVNFLTNISAQIVRTLDEFAQFRDFLLLFQHGVDLVADTFRSHTQVGFEDLTNVHTRRYAQRVQYDVYRSTVFIVRHIFDRVDLRNYTLVTMTACHLVTRLDTAFNRQIYLNNLQHARCQIVALGDFAAFRFEFLLELVFQFVILLSQLFQLILFLFVGQAQLQPAIARQFVEFLSFNATGYQHSTDTAEQTRFEDLQFFRQVFLRLFELHFFDFQRTFVFFYAIASKDLNIDNRTGYTVWYAQRRVFNVRRFLTEDRTQQFFFWGQLSFTFRRYLTNQNVATGHFRTNVNDTGFIQFGESSFTHVRDVSGDLFRPQLGITGHTRQFLNMDGGETVFLNNTLGYEDGVFEVVTIPRHERYAHVLTKRQFTEIGGRTVCQHVAAFYRFTQRHTRHLVDTGVLVRTGVLGQVVDVDTCFTRIHLVFVNFDNDTGSIHVLNDTTTFSNRSYTGVNGNSTFHTRTNQRLISTQSRNGLTLHVRTHQCTVGVIVFQERDQGRTDGYHLLGGYVHVVNLVAAEQAGFAFATASYQVFYEVAFFIQVGVRLGDNVVAFFDSRQIVNFVSYNTVGHFTIRSLKEAVFVSLCVHGQGVDQTDVRTFRGFDWTYATVVSRVYVSNFEACTFTGQTAWAECRDTTFVRNLRQRVVLVHKLRQLAGTEELFHCCGNRLGVDHILRHQGIQIAQRQTLFHRTLYTYQANAELVFRHFANRTDTTVAEVVDIINFAFTVTDIDELFHNINDVVFAQDTGTFDFFAQQRTVELHTTNRRQVIAVFGEEQVLEQAFSSFTSRRLARAHHTVDFYQCAQTVVSWVDT
rpoB	DNA-directed RNA polymerase subunit beta	114	2865	ATA	0	IHLSTAVHVIRLNGEHFLQGVCCAICFQRPHFHLPETLTTELCLTTQRLLSNQAVRTGGTRVHLVVDQVVQFQHVHVTNGYRTLELFTSATVVQADLTRSRQVAKFQQLFNFCFFRTVEDRRCDWHTFAQVFSQTHNFFIAEGTQVNFLTNISAQIVRTLDEFAQFRDFLLLFQHGVDLVADTFRSHTQVGFEDLTNVHTRRYAQRVQYDVYRSTVFIVRHIFDRVDLRNYTLVTMTACHLVTRLDTAFNRQIYLNNLQHARCQIVALGDFAAFRFEFLLELVFQFVILLSQLFQLILFLFVGQAQLQPAIARQFVEFLSFNATGYQHSTDTAEQTRFEDLQFFRQVFLRLFELHFFDFQRTFVFFYAIASKDLNIDNRTGYTVWYAQRRVFNVRRFLTEDRTQQFFFWGQLSFTFRRYLTNQNVATGHFRTNVNDTGFIQFGESSFTHVRDVSGDLFRPQLGITGHTRQFLNMDGGETVFLNNTLGYEDGVFEVVTIPRHERYAHVLTKRQFTEIGGRTVCQHVAAFYRFTQRHTRHLVDTGVLVRTGVLGQVVDVDTCFTRIHLVFVNFDNDTGSIHVLNDTTTFSNRSYTGVNGNSTFHTRTNQRLISTQSRNGLTLHVRTHQCTVGVIVFQERDQGRTDGYHLLGGYVHVVNLVAAEQAGFAFATASYQVFYEVAFFIQVGVRLGDNVVAFFDSRQIVNFVSYNTVGHFTIRSLKEAVFVSLCVHGQGVDQTDVRTFRGFDWTYATVVSRVYVSNFEACTFTGQTAWAECRDTTFVRNLRQRVVLVHKLRQLAGTEELFHCCGNRLGVDHILRHQGIQIAQRQTLFHRTLYTYQANAELVFRHFANRTDTTVAEVVDIINFAFTVTDIDELFHNINDVVFAQDTGTFDFFAQQRTVELHTTNRRQVIAVFGEEQVLEQAFSSFTSRRLARAHHTVDFYQCAQTVVSWVDT
rpoB	DNA-directed RNA polymerase subunit beta	165	2814	CTG	0	LQGVCCAICFQRPHFHLPETLTTELCLTTQRLLSNQAVRTGGTRVHLVVDQVVQFQHVHVTNGYRTLELFTSATVVQADLTRSRQVAKFQQLFNFCFFRTVEDRRCDWHTFAQVFSQTHNFFIAEGTQVNFLTNISAQIVRTLDEFAQFRDFLLLFQHGVDLVADTFRSHTQVGFEDLTNVHTRRYAQRVQYDVYRSTVFIVRHIFDRVDLRNYTLVTMTACHLVTRLDTAFNRQIYLNNLQHARCQIVALGDFAAFRFEFLLELVFQFVILLSQLFQLILFLFVGQAQLQPAIARQFVEFLSFNATGYQHSTDTAEQTRFEDLQFFRQVFLRLFELHFFDFQRTFVFFYAIASKDLNIDNRTGYTVWYAQRRVFNVRRFLTEDRTQQFFFWGQLSFTFRRYLTNQNVATGHFRTNVNDTGFIQFGESSFTHVRDVSGDLFRPQLGITGHTRQFLNMDGGETVFLNNTLGYEDGVFEVVTIPRHERYAHVLTKRQFTEIGGRTVCQHVAAFYRFTQRHTRHLVDTGVLVRTGVLGQVVDVDTCFTRIHLVFVNFDNDTGSIHVLNDTTTFSNRSYTGVNGNSTFHTRTNQRLISTQSRNGLTLHVRTHQCTVGVIVFQERDQGRTDGYHLLGGYVHVVNLVAAEQAGFAFATASYQVFYEVAFFIQVGVRLGDNVVAFFDSRQIVNFVSYNTVGHFTIRSLKEAVFVSLCVHGQGVDQTDVRTFRGFDWTYATVVSRVYVSNFEACTFTGQTAWAECRDTTFVRNLRQRVVLVHKLRQLAGTEELFHCCGNRLGVDHILRHQGIQIAQRQTLFHRTLYTYQANAELVFRHFANRTDTTVAEVVDIINFAFTVTDIDELFHNINDVVFAQDTGTFDFFAQQRTVELHTTNRRQVIAVFGEEQVLEQAFSSFTSRRLARAHHTVDFYQCAQTVVSWVDT
rpoB	DNA-directed RNA polymerase subunit beta	579	2400	ATC	0	IVRTLDEFAQFRDFLLLFQHGVDLVADTFRSHTQVGFEDLTNVHTRRYAQRVQYDVYRSTVFIVRHIFDRVDLRNYTLVTMTACHLVTRLDTAFNRQIYLNNLQHARCQIVALGDFAAFRFEFLLELVFQFVILLSQLFQLILFLFVGQAQLQPAIARQFVEFLSFNATGYQHSTDTAEQTRFEDLQFFRQVFLRLFELHFFDFQRTFVFFYAIASKDLNIDNRTGYTVWYAQRRVFNVRRFLTEDRTQQFFFWGQLSFTFRRYLTNQNVATGHFRTNVNDTGFIQFGESSFTHVRDVSGDLFRPQLGITGHTRQFLNMDGGETVFLNNTLGYEDGVFEVVTIPRHERYAHVLTKRQFTEIGGRTVCQHVAAFYRFTQRHTRHLVDTGVLVRTGVLGQVVDVDTCFTRIHLVFVNFDNDTGSIHVLNDTTTFSNRSYTGVNGNSTFHTRTNQRLISTQSRNGLTLHVRTHQCTVGVIVFQERDQGRTDGYHLLGGYVHVVNLVAAEQAGFAFATASYQVFYEVAFFIQVGVRLGDNVVAFFDSRQIVNFVSYNTVGHFTIRSLKEAVFVSLCVHGQGVDQTDVRTFRGFDWTYATVVSRVYVSNFEACTFTGQTAWAECRDTTFVRNLRQRVVLVHKLRQLAGTEELFHCCGNRLGVDHILRHQGIQIAQRQTLFHRTLYTYQANAELVFRHFANRTDTTVAEVVDIINFAFTVTDIDELFHNINDVVFAQDTGTFDFFAQQRTVELHTTNRRQVIAVFGEEQVLEQAFSSFTSRRLARAHHTVDFYQCAQTVVSWVDT
rpoB	DNA-directed RNA polymerase subunit beta	595	105	ATG	1	MNSRSFATSCCCFSMALILSPIPFAAIPRWVSRI
rpoB	DNA-directed RNA polymerase subunit beta	621	2358	TTG	0	LLLFQHGVDLVADTFRSHTQVGFEDLTNVHTRRYAQRVQYDVYRSTVFIVRHIFDRVDLRNYTLVTMTACHLVTRLDTAFNRQIYLNNLQHARCQIVALGDFAAFRFEFLLELVFQFVILLSQLFQLILFLFVGQAQLQPAIARQFVEFLSFNATGYQHSTDTAEQTRFEDLQFFRQVFLRLFELHFFDFQRTFVFFYAIASKDLNIDNRTGYTVWYAQRRVFNVRRFLTEDRTQQFFFWGQLSFTFRRYLTNQNVATGHFRTNVNDTGFIQFGESSFTHVRDVSGDLFRPQLGITGHTRQFLNMDGGETVFLNNTLGYEDGVFEVVTIPRHERYAHVLTKRQFTEIGGRTVCQHVAAFYRFTQRHTRHLVDTGVLVRTGVLGQVVDVDTCFTRIHLVFVNFDNDTGSIHVLNDTTTFSNRSYTGVNGNSTFHTRTNQRLISTQSRNGLTLHVRTHQCTVGVIVFQERDQGRTDGYHLLGGYVHVVNLVAAEQAGFAFATASYQVFYEVAFFIQVGVRLGDNVVAFFDSRQIVNFVSYNTVGHFTIRSLKEAVFVSLCVHGQGVDQTDVRTFRGFDWTYATVVSRVYVSNFEACTFTGQTAWAECRDTTFVRNLRQRVVLVHKLRQLAGTEELFHCCGNRLGVDHILRHQGIQIAQRQTLFHRTLYTYQANAELVFRHFANRTDTTVAEVVDIINFAFTVTDIDELFHNINDVVFAQDTGTFDFFAQQRTVELHTTNRRQVIAVFGEEQVLEQAFSSFTSRRLARAHHTVDFYQCAQTVVSWVDT
rpoB	DNA-directed RNA polymerase subunit beta	681	2298	GTG	0	VGFEDLTNVHTRRYAQRVQYDVYRSTVFIVRHIFDRVDLRNYTLVTMTACHLVTRLDTAFNRQIYLNNLQHARCQIVALGDFAAFRFEFLLELVFQFVILLSQLFQLILFLFVGQAQLQPAIARQFVEFLSFNATGYQHSTDTAEQTRFEDLQFFRQVFLRLFELHFFDFQRTFVFFYAIASKDLNIDNRTGYTVWYAQRRVFNVRRFLTEDRTQQFFFWGQLSFTFRRYLTNQNVATGHFRTNVNDTGFIQFGESSFTHVRDVSGDLFRPQLGITGHTRQFLNMDGGETVFLNNTLGYEDGVFEVVTIPRHERYAHVLTKRQFTEIGGRTVCQHVAAFYRFTQRHTRHLVDTGVLVRTGVLGQVVDVDTCFTRIHLVFVNFDNDTGSIHVLNDTTTFSNRSYTGVNGNSTFHTRTNQRLISTQSRNGLTLHVRTHQCTVGVIVFQERDQGRTDGYHLLGGYVHVVNLVAAEQAGFAFATASYQVFYEVAFFIQVGVRLGDNVVAFFDSRQIVNFVSYNTVGHFTIRSLKEAVFVSLCVHGQGVDQTDVRTFRGFDWTYATVVSRVYVSNFEACTFTGQTAWAECRDTTFVRNLRQRVVLVHKLRQLAGTEELFHCCGNRLGVDHILRHQGIQIAQRQTLFHRTLYTYQANAELVFRHFANRTDTTVAEVVDIINFAFTVTDIDELFHNINDVVFAQDTGTFDFFAQQRTVELHTTNRRQVIAVFGEEQVLEQAFSSFTSRRLARAHHTVDFYQCAQTVVSWVDT
rpoB	DNA-directed RNA polymerase subunit beta	819	2160	ATG	0	MTACHLVTRLDTAFNRQIYLNNLQHARCQIVALGDFAAFRFEFLLELVFQFVILLSQLFQLILFLFVGQAQLQPAIARQFVEFLSFNATGYQHSTDTAEQTRFEDLQFFRQVFLRLFELHFFDFQRTFVFFYAIASKDLNIDNRTGYTVWYAQRRVFNVRRFLTEDRTQQFFFWGQLSFTFRRYLTNQNVATGHFRTNVNDTGFIQFGESSFTHVRDVSGDLFRPQLGITGHTRQFLNMDGGETVFLNNTLGYEDGVFEVVTIPRHERYAHVLTKRQFTEIGGRTVCQHVAAFYRFTQRHTRHLVDTGVLVRTGVLGQVVDVDTCFTRIHLVFVNFDNDTGSIHVLNDTTTFSNRSYTGVNGNSTFHTRTNQRLISTQSRNGLTLHVRTHQCTVGVIVFQERDQGRTDGYHLLGGYVHVVNLVAAEQAGFAFATASYQVFYEVAFFIQVGVRLGDNVVAFFDSRQIVNFVSYNTVGHFTIRSLKEAVFVSLCVHGQGVDQTDVRTFRGFDWTYATVVSRVYVSNFEACTFTGQTAWAECRDTTFVRNLRQRVVLVHKLRQLAGTEELFHCCGNRLGVDHILRHQGIQIAQRQTLFHRTLYTYQANAELVFRHFANRTDTTVAEVVDIINFAFTVTDIDELFHNINDVVFAQDTGTFDFFAQQRTVELHTTNRRQVIAVFGEEQVLEQAFSSFTSRRLARAHHTVDFYQCAQTVVSWVDT
sodC	Superoxide dismutase	231	117	ATT	0	ILWIKMPACGFCGAGFAIFGGWLAASFGMNMEAMFTGR
sodC	Superoxide dismutase	240	108	ATC	0	IKMPACGFCGAGFAIFGGWLAASFGMNMEAMFTGR
tuf	Elongation factor Tu	55	138	ATT	1	IAKRRPSSIAIGWIRVTTILMLSPGITISTPSGSSMVPVTSVVRK

Offsets are in the antisense direction. Length of putative ORF is in bases. Reading frame is in the antisense direction.

Ten moonlighting genes of *Streptococcus pneumoniae* were found to have putative asORFs with predicted transmembrane domains (Table 16). Six of the ten genes have asORFs containing multiple putative start codons.

**Table 16:** Antisense ORFs containing predicted transmembrane domains, moonlighting genes of *S. pneumoniae.*

Gene	Name	Offset	Length (bases)	Start codon	RF	AA sequence
csd	Cysteine desulfurase	1015	96	ATA	1	ILEGFPWVSCITLVIAEIATADIGVVAALSK
dnaK	Molecular chaperone DnaK	24	378	TAC	0	YDVIACVSCCLCAFCSFLSLLRCCGLFVEFHSKALSFFVQRFKFRFHVVQVVVFLSFLKVIKGSLGSVTFCVEAFTFSFLDCLFSRKDCLVYFITKVYFFFTFLISFSVCFCIFHHAVDFFVSQT
dnaK	Molecular chaperone DnaK	33	369	ATC	0	IACVSCCLCAFCSFLSLLRCCGLFVEFHSKALSFFVQRFKFRFHVVQVVVFLSFLKVIKGSLGSVTFCVEAFTFSFLDCLFSRKDCLVYFITKVYFFFTFLISFSVCFCIFHHAVDFFVSQT
dnaK	Molecular chaperone DnaK	75	327	TTG	0	LSLLRCCGLFVEFHSKALSFFVQRFKFRFHVVQVVVFLSFLKVIKGSLGSVTFCVEAFTFSFLDCLFSRKDCLVYFITKVYFFFTFLISFSVCFCIFHHAVDFFVSQT
dnaK	Molecular chaperone DnaK	216	186	TTG	0	LGSVTFCVEAFTFSFLDCLFSRKDCLVYFITKVYFFFTFLISFSVCFCIFHHAVDFFVSQT
dnaK	Molecular chaperone DnaK	265	171	ATT	1	IVFSVAKIAWSTSLRRSTSSLRFLSASAFASASFIMRSISSSVKPEFDWMTIVCSF
fba	Fructose-bisphosphate aldolase	24	228	TAC	0	YVDTFFNRCLDSFYTVSQEFTWVEEFFLVVFCFVCFVVTSKFTSCVSECDLAFCVNVNFGNTKFDSCLDLLIRNT
fba	Fructose-bisphosphate aldolase	51	201	TTG	0	LDSFYTVSQEFTWVEEFFLVVFCFVCFVVTSKFTSCVSECDLAFCVNVNFGNTKFDSCLDLLIRNT
fusA	Elongation factor G	1119	186	TAC	0	YEWVSHDLEGKSCKWLFVRCWTNFFSVCIWVNTFDCWDVKWAWKVVDNRIKHQLNTFVFEG
gap	NADP-dependent glyceraldehyde-3-phosphate dehydrogenase	19	195	ATG	1	MDLTFVIASMLYLIPCTPAPEPLTPRNGKLSGPRWVLLLMWTVPTSSFSAISKAFLKSFVKTDD
gap	NADP-dependent glyceraldehyde-3-phosphate dehydrogenase	25	189	TTG	1	LTFVIASMLYLIPCTPAPEPLTPRNGKLSGPRWVLLLMWTVPTSSFSAISKAFLKSFVKTDD
gap	NADP-dependent glyceraldehyde-3-phosphate dehydrogenase	37	177	ATC	1	IASMLYLIPCTPAPEPLTPRNGKLSGPRWVLLLMWTVPTSSFSAISKAFLKSFVKTDD
groL	Chaperonin GroEL	20	123	TAC	2	YLDPLLELGLLVLVYWLRLLLLSKSSLLQMLHFVGLNDSL
groL	Chaperonin GroEL	23	120	TTG	2	LDPLLELGLLVLVYWLRLLLLSKSSLLQMLHFVGLNDSL
groL	Chaperonin GroEL	748	288	ATG	1	MAISSSMALRRSPKPGALTATTLKVPRILFKTRVGRASPSTSSAIIKSGRLLWRMLSKSGKISWILEIFLSVIKMYGFSRSATIFSLSVTMYCER
groL	Chaperonin GroEL	772	264	TTG	1	LRRSPKPGALTATTLKVPRILFKTRVGRASPSTSSAIIKSGRLLWRMLSKSGKISWILEIFLSVIKMYGFSRSATIFSLSVTMYCER
pgi	Glucose-6-phosphate isomerase	401	261	ATT	2	IDQVSASQLRSWLKLGRYLFGLFLLIHQPIVSTILRSIEVMAHSLPRSQLHSLYDKGCYERLRIGKLRFQRFCLKCSLCELHSHLP
pgi	Glucose-6-phosphate isomerase	410	252	GTG	2	VSASQLRSWLKLGRYLFGLFLLIHQPIVSTILRSIEVMAHSLPRSQLHSLYDKGCYERLRIGKLRFQRFCLKCSLCELHSHLP
pgi	Glucose-6-phosphate isomerase	437	225	CTG	2	LKLGRYLFGLFLLIHQPIVSTILRSIEVMAHSLPRSQLHSLYDKGCYERLRIGKLRFQRFCLKCSLCELHSHLP
pgi	Glucose-6-phosphate isomerase	738	177	ATC	0	IIWNNKCSPTVSVCFNLNSTLLAVSCCIDTLVSFFLTVFLNQEFFKDTESNRWFSCCT
pgi	Glucose-6-phosphate isomerase	1223	117	TAC	2	YQHQFLYGSIHLLLLLVIACIPLHVVQQICLKLLNNQI
racE	Glutamate racemase	541	132	TTG	1	LISSQTTAVAVLQAMTIILTSLVKRKLTSCQVYSRICSAGRGP
rpoB	DNA-directed RNA polymerase subunit beta	1797	210	TTG	0	LVDPLVTSHDNLLSKGSIFIQTRVSLSYSIFIFFISCQPNNFVRDNTCFTVNLTVWCLNKTIFVQVSVR
rpoB	DNA-directed RNA polymerase subunit beta	1812	195	GTG	0	VTSHDNLLSKGSIFIQTRVSLSYSIFIFFISCQPNNFVRDNTCFTVNLTVWCLNKTIFVQVSVR
sod	Superoxide dismutase	89	117	ATT	2	ISRSKHVPKRPSPRLVFYLLRLVCLGLLLKSLQVSLLC

Offsets are in the antisense direction. Length of putative ORF is in bases. Reading frame is in the antisense direction.

Interestingly, all three organisms scored a transmembrane prediction for antisense ORFs in *rpoB* (DNA-directed RNA polymerase subunit beta) as well as *fba* (fructose-bisphosphate aldolase) and *dnaK* (chaperone DnaK). Notably, of the 89 total transmembrane-domain predictions that occurred across the three model organisms, 49 (55.1%) are in reading frame + 1, with only 8 in the + 2 reading frame. Most of the putative antisense ORFs in Tables 14, 15, and 16 are small (with median lengths ranging from 65 amino acids in *Streptococcus* to 92 in *E. coli*). This is not unexpected, given recent work^7^ showing that genes encoding small (< 100 AA) proteins may account for 16% (± 9%) of all proteins in bacteria, with many of such genes involved in membrane proteins, and some encoded in antisense^8^. However, not all transmembrane-containing asORFs are small. The largest putative transmembrane-containing antisense ORF that we found in *E. coli* is 2892 bases long and occurs in *rpoB*.

## Discussion

In this study, we looked at forward and reverse-complement purine bias in base one of codons (and anticodons), and we found that most moonlighting genes (not only in *M. tuberculosis*, but also in *E. coli* and *Streptococcus pneumoniae*) score well above the CDS-genome average for forward and reverse purine bias. Based on this finding, we adopted a provisional assumption that antisense translation products might be encoded in moonlighting genes. This led us to hypothesize that codons meeting the pattern RNY (purine, any base, pyrimidine) might exist in relative abundance in moonlighting genes. And indeed, we found that by scoring all of an organism’s genes according to “RNY content,” we could enrich for moonlighting genes: we obtained fold-enrichments of 2.29–2.58, at hypergeometric expectations of zero to 0.002, in the three model organisms. We reasoned that if information is being encoded bidirectionally in at least some portions of moonlighting genes, a consequence of this would be that degenerate codon bases (base 1, base 3) would need to accommodate the informational load in such a way that information is essentially multiplexed. To test this possibility, we came up with a heuristic based on the idea that codon information can be encoded differentially along RY and SW axes. (RY refers to the IUPAC ambiguity axis of purine versus pyrmidine; SW refers to the axis of GC versus AT.) We assessed base-1 Shannon entropy in the RY axis, and base-3 Shannon entropy in that axis, for each gene; using these numbers, we created a vector [HRY1, HRY3]. In like manner, we created a vector [HSW1, HSW3] for the SW entropies of bases 1 and 3. We then calculated the dot product of the vectors so created, and used a metric of *1−dotProduct* to sort genes. This metric produced a substantial enrichment for moonlighting genes in *M. tuberculosis*, and the combination of the RNY metric plus the dot-product metric produced significant moonlighting-gene enrichments in all three organisms.

Encouraged by these findings, we looked for antisense open reading frames (asORFs) in moonlighting genes, and found 142 of them in 81/86 moonlighting genes in the three model organisms. We predicted, on purely theoretical grounds, that most antisense transcripts would be in the + 1 reading frame; and indeed this turned out to be the case (90 of 142 asORFs were + 1). We also predicted asORFs in the + 1 frame would contain mostly pyrimidines in base two of codons. This was also the case. The average Y2 (pyrimidine, base 2) content of asORFs in reading frame + 1 ranged from 0.6309 to 0.7046. Since a codon with pyrimidine in base 2 usually specifies a non-polar amino acid, we anticipated that moonlighting-gene asORFs might encode membrane proteins. When we checked the translation products of the putative asORFs for transmembrane domains, 89 such protein products were predicted (by the CCTOP prediction server at https://cctop.ttk.hu/) to contain transmembrane domains.

**Figure 5:**
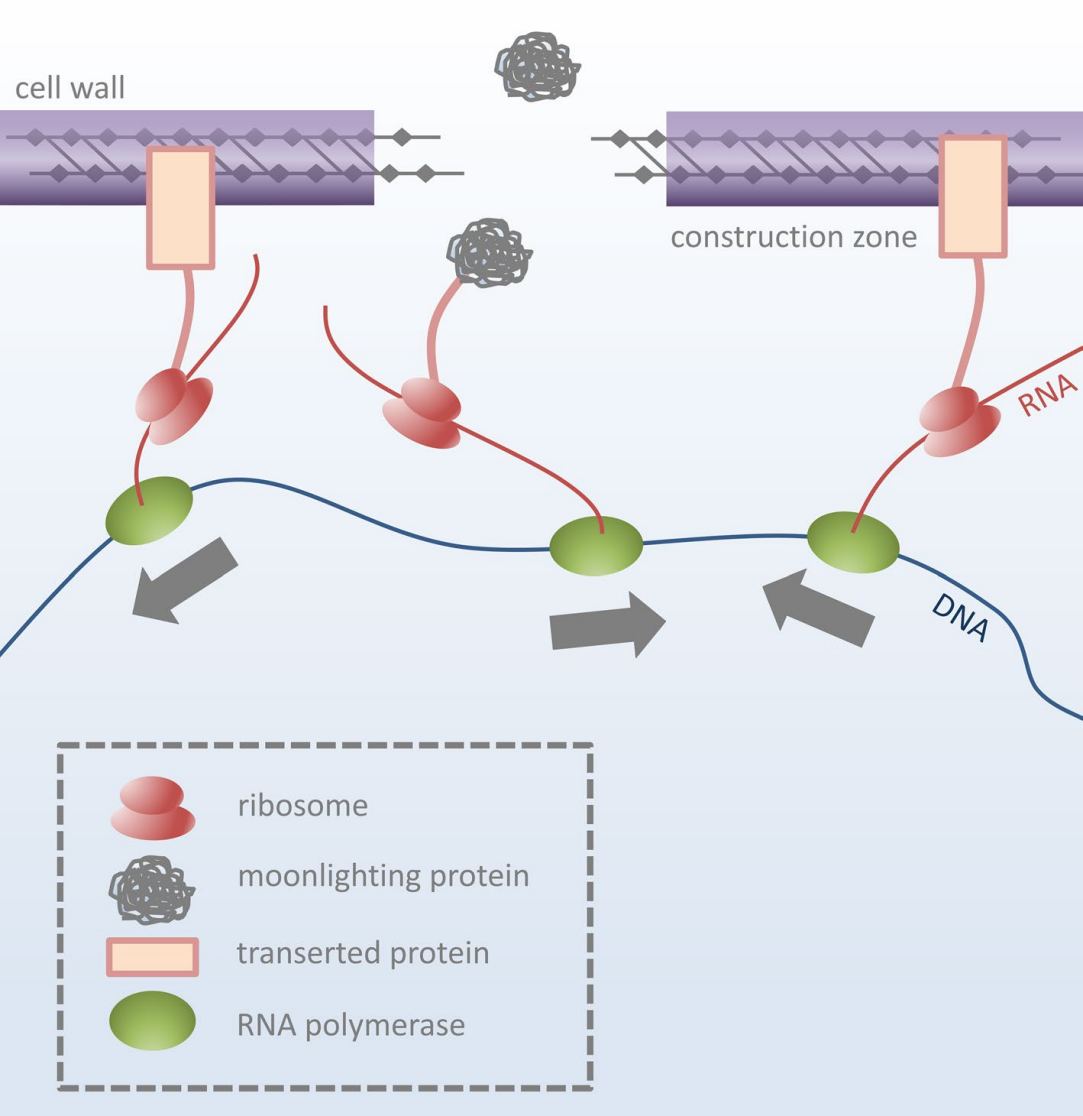
A possible scenario involving translation of a moonlighting gene. Transcription occurs bidirectionally (see green orbs, above, representing RNA polymerase). “Wrong-way” RNAPs, at the ends of the moonlighting gene, produce antisense products containing membrane-friendly polypeptides that associate with the membrane, possibly via transertion (but possibly via some other mechanism). The membrane-friendly antisense products provide firm anchors to the DNA. Intensive cell wall construction is underway in the area; this means there are gaps in the wall. The moonlighting gene, anchored at each end by transertion tethers, is held in close physical proximity to the open section of wall. A newly produced moonlighting protein, when it detaches from its ribosome, easily passes through the gap in the cell wall. It may, in fact, have nowhere else to go. See text for further “Discussion”.

Based on these findings, and based on our finding that moonlighting genes tend to co-locate with genes involved in cell wall or cell membrane construction (see the section “*Location of Moonlighting Genes*” further above), we propose the model for moonlighting shown in Fig. 5, which we call the THX1138 Model, named after the protagonist of George Lucas’s first motion picture (“THX1138”), in which the hero—trapped in a subterranean dystopia—escapes to the surface of the planet, where he sees sunlight for the first time.

Bidirectional transcription of the moonlighting gene causes nascent antisense proteins to be produced, which stick to the membrane. (This hypothesis is based on our finding that the antisense proteins in question often contain putative transmembrane domains). In growth phase, translation is transcriptionally coupled, so that if the nascent antisense protein(s) sticks to the membrane as it is being manufactured, the DNA is essentially tethered to the membrane. Genes immediately upstream and downstream of the moonlighting gene may also have antisense transertion tethers, formed through the same mechanism. (Evidence for transertion tethering of the kind mentioned here has been documented for a number of bacterial species; see the review by Woldringh^8^). We hypothesize that anchoring of DNA to the membrane in this fashion is (possibly) a widespread phenomenon, perhaps involving hundreds of genes. (This view is consistent with recent research on small proteins in bacteria^9^, many of which have ORFs that exist in overlapping reading frames^10^, often in antisense^11^). In the area between the ends of the gene, intensive cell wall construction may be occurring, and we hypothesize that there are areas where sizable (~ 10–30 nm) gaps exist—areas where bridging of the gap by transertionally tethered DNA may, in fact, be an essential structural reinforcement to prevent the weakened wall from opening up catastrophically. Meanwhile, gaps in the under-construction wall are large enough to allow whole proteins to pass through unrestricted, pushed out forcibly under turgor pressure.

Regardless of whether antisense proteins are produced, the escape of moonlighting proteins to the surface of the cell can be explained rather simply by the fact that production of moonlighting proteins occurs in close proximity to areas of intensive cell wall and cell membrane construction. A straightforward leakage hypothesis is not only warranted, but compelling—and easily explains why no secretion systems have ever been implicated in ECP release. “Non-classical secretion” is simply propitious leakage. More complicated explanations should not be pursued until simple ones have been ruled out (Occam’s Razor). Parsimony dictates that we should entertain a leakage theory before others; the burden of proof is on those who insist on more complex explanations. Given the 5–30 atmospheres of turgor pressure that exist inside a bacterial cell^12^, gene products produced near a “hole in the wall” might very well be forced, violently, through the hole, like a passenger blown out an airliner window after explosive decompression at 30,000 feet.

The THX1138 Model (propitious leakage aided by forced proximity via the action of membrane-bound polysomes) explains a number of aspects of “moonlighting” that have managed to elude explication for 30 years:

1. It explains why functionally unrelated enzymes (glycolytic enzymes, chaperones, elongation factors, superoxide dismutases, etc.) are involved. They have something in common: their antisense products are rich enough in non-polar amino acids to stick to the membrane.
2. It explains how ECP-type moonlighting genes achieve excretion: Aided by turgor pressure, they are squeezed out through holes in the under-construction membrane/wall complex, as a consequence of being produced in exactly the right location for this to happen, with non-polar antisense proteins anchoring the gene to the membrane in particularly porous areas.
3. It explains why some proteins, from the same operon, are excreted while others are not. Some glycolysis genes, for example, might produce antisense products that stick to the cell membrane, while others have no antisense products at all (or products that are too short, or too polar, to serve in the “stick to the membrane” role).
4. It explains why Boël et al.^13^ were able, by modifying the 3’ end of the GAPDH gene in streptococcus, to prevent excretion of GAPDH. Modifying the 3’ end of the gene could easily change the membrane-binding properties of a gene’s antisense product. It could reduce or eliminate the translatability of antisense product(s).
5. It explains why moonlighting genes are located next to membrane and cell wall construction genes: they (or rather, their antisense products) play a role in holding the membrane together while it is being built.

Our theory can be seen as encompassing two hypotheses: one is that leakage of moonlighting proteins occurs past areas of active cell wall/membrane construction. The other is that such leakage (if it occurs) is facilitated by membrane-friendly antisense proteins, which (during co-transcriptional translation) essentially tether moonlighting genes to the cell wall/membrane. While it is possible that leakage of moonlighting proteins past areas of active cell-wall/membrane construction may be occurring without help from antisense-protein-related tethering of DNA to the inner membrane, we believe the tethers (if they exist) may, in fact, be essential in “holding the door open.”

### Evolutionary implications

Hundreds of genes co-enrich with moonlighting genes when a scoring metric is used that assumes bidirectionality of transcription and translation. Based on our enrichment experiments, it appears likely that many genes encode information on both DNA strands (in at least some sections). This situation is, of course, made possible by the degeneracy of the genetic code. Degeneracy is also what allows most point mutations to remain “neutral” (via synonymous codons). But in a region of bidirectional information flow, neutrality is necessarily reduced. In a configuration where codons and anticodons are offset by one base, with anticodons in reading frame + 1, neutrality is preserved in base 3, since in this configuration base 3 of codons will overlap base 3 of anticodons (Fig. 4). However, in other alignments, base 3 will overlap an information-rich (degeneracy-poor) base, and synonymous mutations will necessarily be rarer. For large regions of applicable genes, there may not be such a thing as a “neutral mutation.” From theoretical considerations, we can confidently predict that a + 1 offset of the antisense ORF will be the most neutrality-conserving alignment, since it puts base 3 of codons in alignment with base 3 of anticodons. For organisms with relatively high codon Shannon entropies, which is to say organisms having an average G + C content close to 50%, this is the alignment that gives the most protection against non-synonymous mutations. Organisms with significantly higher or lower GC content will have more “informational headroom” in their codons (because of GC or AT redundancy) and may thus be able to tolerate antisense reading frame offsets of zero or + 2. On this basis, we would predict that very-low-GC organisms might not have asORFs that are biased in favor of the + 1 antisense offset; other offsets will also be utilized. Even so, genes with significant two-way information content will (regardless of ORF framing) be less tolerant of point mutations and can therefore be expected to evolve slowly, appearing as “highly conserved genes” undergoing purifying selection. Kimura’s original “neutral theory”^14^ did not focus on point mutations directly; it merely posited that most “mutations” have little to no effect on phenotypes. Nevertheless the existence of bidirectional information in at least some genes constitutes an important footnote to any discussion of mutation-based evolution. Synonymous versus non-synonymous mutation rates, transversion/transition ratios, and other dynamics will need to be considered carefully in light of the bidirectionality (or non-bidirectionality) of various regions of genes. The demands of bidirectional evolution may place unusual constraints on codon composition.

### Predictions of the model

Because our model allows us to construct metrics that consistently enrich for moonlighting genes, it allows for prediction of moonlighting functionality in genes that have not yet received such assignments. In Table 17, we present nine such predictions, representing genes that consistently co-enrich with moonlighting genes in all three of our model organisms. We expect some or all of these nine genes to be “moonlighters” of the ECP type. These are genes that consistently occur in our enrichment experiments, and do so across all three model organisms.

**Table 17:** Predicted moonlighting proteins.

Gene	Function
rpoC	DNA-directed RNA polymerase subunit beta'
gyrA	DNA gyrase subunit A
gyrB	DNA topoisomerase (ATP-hydrolyzing) subunit B
ligA	NAD-dependent DNA ligase LigA
typA	Translational GTPase TypA
ptsP	Phosphoenolpyruvate–protein phosphotransferase
infB	Translation initiation factor IF-2
purH	Bifunctional phosphoribosylaminoimidazolecarboxamide formyltransferase/IMP cyclohydrolase
aspS	Aspartate–tRNA ligase

These are genes that consistently occur in moonlighting-gene enrichment experiments, in all three model organisms (*M. tuberculosis, E. coli*, and *S. pneumoniae*). Their consistent co-enrichment suggests that they are, in fact, moonlighters.

While it is obviously impossible for us to predict what the secondary function of any of these genes might be, nevertheless we would, at a minimum, expect the gene products in question to exist extra-cellularly (either on the surface of cells, or in the culture supernatant), via the “propitious leakage” mechanism.

### Limitations of the current study

One important limitation of our study is that we did not attempt to look for antisense ORFs that span gene boundaries. We would expect some such ORFs to exist, since roughly 15% of genes in each of our model organisms are leaderless (adjoining; in some cases, overlapping) genes that may be transcribed polycistronically. We also did not attempt a comprehensive search for intra-gene promoters in the antisense strands of moonlighting genes. Antisense intra-gene promoters are present in about 11% of *M. tuberculosis* genes (based on our unpublished data), and we believe these promoters may play a role in modulating the expression of asORFs. Upstream antisense promoters might also exist in the neighbors of moonlighting genes. This is an area for further research.

Our enrichments for moonlighting genes, though moderately successful (with fold-enrichments of 2.0–3.0), did not “find” all moonlighting genes. So it’s fair to ask, why not? Why didn’t all moonlighters enrich? We believe there are several possible answers. First, our metrics did not take into account effects that might involve antisense ORFs that cross gene boundaries (as mentioned above), and it is possible such effects could be important. Moonlighting is, after all, we believe, a hitchhiking phenomenon, arising from the tendency of certain chaperones, metabolic genes, and others to “ride the coat-tails” of cell-wall synthesis genes in the course of many syntenic crossover events and/or other gene relocation events. It would make sense if moonlighting is related not only to antisense products arising from moonlighting genes themselves, but nearby neighbor genes as well. It may also be that some moonlighting genes have silent secretion partners in the form of hypothetical proteins. This could be particularly true for *M. tuberculosis*, where enrichment stats were generally weaker than for *E. coli* or *S. pneumonia.* In *M. tuberculosis*, more than in the other two organisms, moonlighting genes tend to cluster near hypothetical protein genes, as a result of that organism having 26.9% hypothetical protein genes versus 5.5% for *E. coli* and 9.7% for *Streptococcus* But the answer to the question “Why didn’t all moonlighting genes enrich?” might be simpler still. Our enrichment probes were effective in enriching many other categories of genes (e.g. genes for ribosomal proteins, cell wall biogenesis, fatty acid synthesis, transporters, permeases, and others), suggesting that antisense ORFs with the potential to produce small membrane proteins might be extremely common, involving perhaps ~ 20% of all genes. Our techniques enriched all of those genes, making dilution of our moonlighting harvest inevitable.

More generally, a limitation of the current study is that we did not undertake wet-lab investigations to determine if antisense ORFs are actually translated, nor did we search the ribosome-profiling data online to see if any of these ORFs have been uncovered in high-throughput profiling. We identified antisense ORFs using what we feel is industry standard ORF-calling logic, but we are unable to make (and do not make) any claim, one way or the other, on whether these ORFs are, in fact, translated in vivo. We invite other researchers to pursue this area further.

## Conclusions

In this study, we found that moonlighting genes of three model organisms (*Mycobacterium tuberculosis* H37Rv, *Escherichia coli* NCTC11775, and *Streptococcus pneumoniae* NCTC11032) tend to co-locate, on the genome, near genes involved in cell wall biogenesis, secretion, and inner or outer membrane synthesis. We were able to create a simple bioinformatics probe that quantifies the potential for reverse transcription and antisense translation, and found that such a probe allows us to discover moonlighting genes by means of a straightforward enrichment assay technique. Based on theoretical considerations, we predicted that if any antisense open reading frames existed in moonlighting genes, they would most likely exist in reading frames zero or + 1; frame + 2 would probably not be well utilized. We also predicted that any ORFs found to be in reading frame + 1 would encode proteins with a high percentage of nonpolar amino acids. We were able to validate these predictions. When we looked for antisense ORFs in moonlighting genes, we found 142 putative ORFs across the three model organisms, 90 of which were in reading frame + 1. Moreover, the 90 antisense ORFs of reading frame + 1 had comparatively high non-polar amino acid content. When we checked the putative translation products of antisense ORFs using the CCTOP transmembrane prediction server, we found that seven translation products of *M. tuberculosis*, fifteen products from *E. coli*, and ten products from *S. pneumoniae* contained predicted transmembrane domains. Most of the remaining products are expected to be membrane proteins based on high nonpolar amino acid content. Based on these findings, we presented a model that proposes a role for antisense membrane proteins in binding moonlighting genes to the bacterial inner membrane, in areas of active cell wall construction. Because moonlighting proteins are produced in “forced proximity” to porous areas of new cell wall, and because turgor pressure in bacterial cells is extreme (5–30 atmospheres), escape of moonlighting proteins to the exterior of the cell, through gaps in the wall, is (we believe) unavoidable. Our model allows us to predict that certain proteins (which have not yet been found to be moonlighters) will likely have an extracellular role. Thus, we made specific predictions for nine genes: *rpoC, gyrA, gyrB, LigA, typA, ptsP, infB, purH,* and *aspS*.

### Supplementary Information

Supplementary Information.

## Data Availability

The datasets generated and/or analysed during the current study are available in the Github repository, [https://github.com/kasmanethomas/moonlighting] and [https://cctop.ttk.hu/].
