# Supplementary material for: Moonlighting genes harbor antisense ORFs that encode potential membrane proteins
Source: Sci Rep. 2023 Aug 3;13:12591. doi: 10.1038/s41598-023-39869-x (PMC10400600; doi:10.1038/s41598-023-39869-x)
Supplement: Supplementary file 1 — Supplementary Information. [file 41598_2023_39869_MOESM1_ESM.pdf]

Supplement A

Figure 1 shows purine content of codon bases, versus genome G+C content, for N = 159 bacteria. The raw data are shown further below. The columns are (from left to right):

1. Average CDS-genome G+C content.
2. Average purine (A+G) content for base 1 of all codons.
3. Purine content, base 2.
4. Purine content, base 3.
5. Organism name (genus, species, and strain). The “id” identifier is the private database key for the genome assigned by https://genomevolution.org/.

The numbers in Figure 1 were imputed from codon-table statistics. The codon tables were obtained by viewing all organisms in a single GenomeList web page (https://genomevolution.org/CoGe/GenomeList.pl) with Get All chosen under the Codon Usage column (so as to expose codon tables). The organisms can be accessed using GenomeList by going to:
https://genomevolution.org/CoGe/GenomeList.pl?dsgid=7026,9667,8178,3440,13788,21269,8203,9731,21488,21461,9753,9755,3504,13389,9767,16937,9784,21867,21476,21076,9812,21015,9893,21897,14715,11995,21918,21181,21276,21598,9974,7236,9977,21151,21742,7254,3763,3776,15637,7291,21946,14691,13918,3853,3886,21594,3891,3902,20904,21040,20909,21962,18283,19085,15441,3959,16247,3969,3973,10161,15677,7336,20948,3987,7346,21980,7347,18233,19041,11312,15114,3994,15903,7360,21994,21384,11003,21614,13405,12413,21765,4096,4103,16569,4105,4111,7407,4124,7410,13551,8443,7454,4262,7481,10459,4305,17281,4315,21187,21185,4319,8501,4321,19351,18666,17556,8511,17551,4328,4331,15659,10811,20707,4397,22176,8581,9584,13596,19070,20540,18839,4507,21634,15966,4518,17777,4519,14167,13321,11624,21521,14035,4593,21708,11777,14193,21529,21498,21209,20667,21197,17938,22245,4683,18688,4696,19574,21586,19392,4735,21716,4756,21282,16546,4765,17066,4792,19304,11595,16590,18583,4815,4816,16043,4818,19097,18302,8021,22292,15526,4822,16535,17263,4841,7723,11563,21251,16539,3297,21479,16387,21272,3179,7742,8857,3167,3150,16557,2338,7775,21337,15776,7780,8915,17264,19037,2238,11326,8984,2124,21571,15707,2110,16636,2096,18743,19370,21734,2064,15520,21744,21514,2043,10356,7901,1970,14980

Duplicate species were removed, and any organisms with empty codon tables were removed.

Organisms were chosen based on being uniformly distributed across a wide G+C spectrum.

For each organism, codon percentages were summed in base-position-specific fashion to derive

the relevant statistics. For example, all codons beginning with ‘G’ had their numbers summed to arrive at the base-1 guanine content. A similar summation was done for adenine, cytosine, and thymine across each base position.

0.4811 0.5748 0.4748 0.4617 Acaryochloris marina strain MBIC11017 [id: 7026]

0.5714 0.6258 0.4857 0.4831 Acidaminococcus fermentans strain DSM 20731 [id: 9667]

0.6904 0.5876 0.4507 0.4618 Acidovorax avenae subsp. citrulli strain AAC00-1 [id: 3440]

0.4308 0.6264 0.4989 0.4326 Aerococcus urinae strain ACS-120-V-Col10a [id: 13788]
This look so good !
0.6254 0.587 0.4718 0.4391 Aeromonas hydrophila strain ML09-119 [id: 21269]

0.4561 0.6054 0.4789 0.4718 Aggregatibacter actinomycetemcomitans D11S-1 [id: 8203]

0.6079 0.6125 0.4438 0.4203 Agrobacterium radiobacter strain K84 [id: 9731]

0.5786 0.6108 0.4847 0.45 Alistipes finegoldii strain DSM 17242 [id: 21488]

0.4856 0.6492 0.4703 0.5027 Anaerobaculum mobile strain DSM 13181 [id: 21461]

0.3561 0.6719 0.4988 0.5279 Anaerocellum thermophilum strain DSM 6725 [id: 9753]

0.3516 0.6887 0.508 0.4973 Anaerococcus lactolyticus strain ATCC 51172 [id: 9755]

0.5438 0.5756 0.4551 0.4708 Anaerolinea thermophila strain UNI-1 [id: 13389]

0.4981 0.6415 0.4733 0.4988 Anaplasma marginale strain Florida [id: 9767]

0.2723 0.6772 0.4943 0.5173 Arcobacter butzleri ED-1 [id: 16937]

0.4504 0.5863 0.4841 0.4673 Arthrospira maxima strain CS-328 [id: 9784]

0.4321 0.6123 0.4704 0.4347 Atopobium vaginae strain DSM 15829 [id: 21867]

0.6915 0.6008 0.4482 0.4453 Azospirillum brasilense strain Sp245 [id: 21476]

0.4671 0.6361 0.4831 0.5046 Bacillus amyloliquefaciens strain Y2 [id: 21076]

0.3591 0.6439 0.4841 0.5379 Bacillus anthracis strain CDC 684 [id: 9812]

0.4648 0.6146 0.4767 0.4804 Bacillus subtilis BEST7613 strain PCC 6803 [id: 21015]

0.359 0.6445 0.4846 0.5379 Bacillus thuringiensis serovar andalousiensis strain BGSC 4AW1 [id: 9893]

0.4269 0.6244 0.5047 0.4795 Bacteroides dorei strain 5_1_36/D4 [id: 21897]

0.4288 0.6256 0.5044 0.4765 Bacteroides ovatus strain 3_8_47FAA [id: 14715]

0.4037 0.6065 0.4659 0.4519 Bartonella quintana strain RM-11 [id: 21181]

0.5039 0.6142 0.4763 0.4541 Bdellovibrio bacteriovorus strain Tiberius [id: 21276]

0.7304 0.6018 0.4316 0.4475 Blastococcus saxobsidens strain DD2 [id: 21598]

0.5748 0.5932 0.4719 0.4692 Blastopirellula marina strain DSM 3645 [id: 9974]

0.4568 0.6467 0.5043 0.5347 Blautia hydrogenotrophica (Ruminococcus hydrogenotrophicus DSM 10507) strain DSM 10507 [id: 7236]

0.6811 0.5859 0.4498 0.4526 Bordetella pertussis strain 18323 [id: 21151]

0.2899 0.665 0.4977 0.4794 Borrelia miyamotoi strain LB-2001 [id: 21742]

0.7209 0.5994 0.432 0.4236 Brachybacterium faecium strain DSM 4810 [id: 7254]

0.5826 0.6137 0.4523 0.4305 Brucella ovis strain ATCC 25840 [id: 3763]

0.2741 0.6327 0.4916 0.4993 Buchnera aphidicola (Acyrthosiphon pisum) strain 5A [id: 3776]

0.3809 0.6886 0.5045 0.5006 Butyrivibrio crossotus strain DSM 2876 [id: 15637]

0.3657 0.6722 0.4988 0.5257 Caldicellulosiruptor lactoaceticus strain 6A [id: 14691]

0.359 0.6728 0.489 0.5041 Calditerrivibrio nitroreducens strain DSM 19672 [id: 13918]

0.4004 0.6813 0.506 0.473 Campylobacter concisus strain 13826 [id: 3853]

0.3821 0.6264 0.4919 0.4435 Candidatus Cloacamonas acidaminovorans [id: 3886]

0.5904 0.5963 0.4608 0.4844 Candidatus Methylomirabilis oxyfera [id: 21594]

0.2979 0.6729 0.492 0.5035 Candidatus Pelagibacter ubique strain HTCC1062 [id: 3891]

0.4239 0.6442 0.4815 0.5054 Carboxydothermus hydrogenoformans strain Z-2901 [id: 3902]

0.4164 0.5751 0.4575 0.469 Chlamyda trachomatis (i) strain L2/434/Bu; i [id: 20904]

0.3964 0.5841 0.4652 0.4633 Chlamydia psittaci (Chlamydophila psittaci 6BC) strain 6BC [id: 21040]

0.4181 0.5758 0.4585 0.4702 Chlamydia trachomatis (s)/852 strain RC-F; s/852 [id: 20909]

0.3786 0.6488 0.4993 0.4877 Chryseobacterium gleum strain ATCC 35910 [id: 21962]

0.2914 0.697 0.507 0.5513 Clostridium botulinum A strain Hall [id: 3959]

0.3815 0.6754 0.498 0.5097 Clostridium cellulolyticum strain ATCC 35319; H10 [id: 16247]

0.296 0.6937 0.5021 0.5486 Clostridium difficile strain CIP 107932 [id: 3973]

0.5296 0.6193 0.488 0.4486 Clostridium methylpentosum strain DSM 5476 [id: 7346]

0.3777 0.6707 0.4981 0.5081 Clostridium papyrosolvens strain DSM 2782 [id: 7347]

0.2907 0.6989 0.5146 0.5415 Clostridium perfringens B strain ATCC 3626 [id: 3994]

0.6283 0.5968 0.4588 0.4496 Comamonas testosteroni strain KF-1 [id: 7360]

0.3234 0.6584 0.506 0.4872 Coprobacillus sp. strain 8_2_54BFAA [id: 21994]

0.4408 0.6586 0.4975 0.4926 Coprococcus catus strain GD/7 [id: 21384]

0.5339 0.6303 0.5072 0.4967 Corynebacterium tuberculostearicum strain B146 [id: 11003]

0.4102 0.599 0.4785 0.4878 Crinalium epipsammum strain PCC 9333 [id: 21614]

0.3879 0.5996 0.4856 0.4653 Cyanothece sp. strain ATCC 51472 [id: 13405]

0.4229 0.6217 0.4767 0.4803 Cycloclasticus zancles strain 7-ME [id: 21765]

0.6723 0.5894 0.4613 0.4556 Deinococcus radiodurans strain R1 [id: 4096]

0.5723 0.621 0.4643 0.4484 Desulfococcus oleovorans strain Hxd3 [id: 4103]

0.4323 0.6432 0.4822 0.4858 Desulfotomaculum reducens strain MI-1 [id: 16569]

0.6748	0.5971	0.4487	0.452	Desulfovibrio vulgaris strain Miyazaki F [id: 4111]
0.526	0.6172	0.4842	0.4673	Dyadobacter fermentans strain DSM 18053 [id: 7407]
0.3098	0.6507	0.489	0.5035	Ehrlichia canis strain Jake [id: 4124]
0.5718	0.5909	0.4765	0.4234	Eikenella corrodens strain ATCC 23834 [id: 7410]
0.5835	0.597	0.4639	0.4685	Enterobacter cloacae strain SCF1 [id: 13551]
0.5463	0.5903	0.4735	0.4741	Erwinia amylovora strain ATCC 49946 [id: 8443]
0.5188	0.6007	0.4706	0.4718	Escherichia coli B strain REL606 [id: 7454]
0.3754	0.6512	0.497	0.4823	Flavobacteriales bacterium strain HTCC2170 [id: 4262]
0.5283	0.6096	0.4763	0.5097	Geobacillus kaustophilus strain HTA426 [id: 4305]
0.5308	0.6078	0.4775	0.5113	Geobacillus thermoleovorans strain CCB_US3_UF5 [id: 17281]
0.6032	0.6179	0.4662	0.4307	Geobacter metallireducens strain GS-15 [id: 4315]
0.6161	0.6152	0.4599	0.4308	Geobacter sulfurreducens strain KN400 [id: 21187]
0.6157	0.6148	0.4606	0.4316	Geobacter sulfurreducens strain PCA [id: 21185]
0.5535	0.6294	0.4722	0.4505	Geobacter uraniireducens strain Rf4 [id: 4319]
0.7413	0.5998	0.4295	0.4425	Geodermatophilus obscurus strain DSM 43160 [id: 8501]
0.6284	0.578	0.4624	0.4425	Gloeobacter violaceus strain PCC 7421 [id: 4321]
0.684	0.6133	0.4364	0.4412	Gordonia araii strain NBRC 100433 [id: 17556]
0.6738	0.6107	0.434	0.4311	Gordonia bronchialis strain DSM 43247 [id: 8511]
0.6274	0.612	0.432	0.4531	Gordonia effusa strain NBRC 100432 [id: 17551]
0.3726	0.6551	0.504	0.4819	Gramella forsetii strain KT0803 [id: 4328]
0.3875	0.61	0.4789	0.4888	Haemophilus ducreyi strain 35000HP [id: 4331]
0.6993	0.594	0.4693	0.4169	Haliangium ochraceum strain DSM 14365 [id: 15659]

0.6106 0.6347 0.4713 0.4393 Halogeometricum borinquense DSM 11551 [id: 10811]

0.393 0.6377 0.5079 0.4652 Helicobacter pylori (Helicobacter pylori SAfr7) strain SouthAfrica7 [id: 20707]

0.5517 0.612 0.4556 0.4685 Janthinobacterium sp. strain Marseille [id: 4397]

0.5627 0.5977 0.4663 0.4606 Klebsiella oxytoca strain 10-5243 [id: 22176]

0.7086 0.6003 0.4388 0.4671 Kribbella flavida strain DSM 17836 [id: 8581]

0.5513 0.57 0.4741 0.4551 Ktedonobacter racemifer DSM 44963 [id: 9584]

0.3887 0.6487 0.4983 0.4388 Lactobacillus acidophilus strain 30SC [id: 13596]

0.3942 0.6285 0.4927 0.4658 Lactobacillus reuteri strain MM4-1A [id: 19070]

0.3677 0.6361 0.4789 0.4689 Lactococcus lactis subsp. cremoris strain A76 [id: 20540]

0.3467 0.6076 0.4692 0.4812 Lawsonia intracellularis strain PHE/MN1-00 [id: 18839]

0.4485 0.6009 0.4796 0.4485 Leptolyngbya sp. PCC 7376 [id: 21634]

0.5501 0.5451 0.485 0.4573 Leptolyngbya valderiana strain BDU 20041 [id: 15966]

0.5463 0.5858 0.4712 0.4421 Leptonema illini strain DSM 21528 [id: 17777]

0.39 0.6019 0.475 0.4833 Leptospira biflexa serovar Patoc strain Ames; Patoc 1 [id: 4519]

0.3727 0.6343 0.4612 0.4912 Leuconostoc gasicomitatum LMG 18811 strain type LMG 18811 [id: 14167]
0.4438 0.592 0.4873 0.4769 Lyngbya majuscula strain 3L [id: 13321]

0.6348 0.6145 0.4435 0.4321 Mesorhizobium australicum strain WSM2073 [id: 21521]

0.6324 0.6164 0.4429 0.4315 Mesorhizobium ciceri biovar biserrulae strain WSM1271 [id: 14035]

0.5655 0.6051 0.4694 0.4544 Methylobacillus flagellatus strain KT [id: 4593]

0.4817 0.5998 0.4741 0.4694 Methylophaga sp. strain JAM7 [id: 21708]

0.4722 0.6022 0.4752 0.4858 Microcoleus vaginatus strain FGP-2 [id: 11777]

0.6902 0.6092 0.4335 0.4547 Mycobacterium chubuense strain NBB4 [id: 21529]

0.6848 0.6035 0.4395 0.4587 Mycobacterium indicus pranii MTCC 9506 [id: 21498]

0.6545 0.6104 0.4387 0.4638 Mycobacterium smegmatis strain JS623 [id: 21209]

0.6586 0.6086 0.4396 0.4645 Mycobacterium tuberculosis = ATCC 35801 strain ATCC35801; Erdman [id: 20667]

0.3192 0.6419 0.5024 0.4645 Mycoplasma gallisepticum strain F [id: 21197]

0.3492 0.6514 0.4975 0.5119 Myroides odoratimimus strain CIP 101113 [id: 17938]

0.6911 0.5783 0.4517 0.4914 Myxococcus xanthus strain DK 1622 [id: 4683]

0.535 0.6146 0.483 0.445 Neisseria gonorrhoeae strain NCCP11945 [id: 4696]

0.6859 0.6016 0.4316 0.4568 Nocardia brasiliensis ATCC 700358 strain HUJEG-1 [id: 19574]

0.6882 0.6057 0.4342 0.4415 Nocardia cyriacigeorgica strain GUH-2 [id: 21586]

0.4231 0.5985 0.4766 0.4775 Nostoc sp. PCC 7120 (Anabaena sp. PCC 7120) strain PCC7120 [id: 19392]

0.6552 0.6121 0.4516 0.4305 Novosphingobium aromaticivorans strain DSM 12444 [id: 4735]

0.3573 0.6436 0.4845 0.5282 Oceanobacillus kimchii strain X50 [id: 21716]

0.3154 0.6457 0.5011 0.504 Orientia tsutsugamushi strain Ikeda [id: 4756]

0.4618 0.6167 0.4802 0.5011 Paenibacillus polymyxa strain M1 [id: 21282]

0.4603 0.6185 0.4933 0.4788 Parabacteroides distasonis strain ATCC 8503 [id: 16546]

0.5601 0.5938 0.4741 0.4399 Pelobacter carbinolicus strain DSM 2380 [id: 4792]

0.4599 0.6158 0.4647 0.445 Polynucleobacter necessarius strain STIR1 [id: 4815]

0.494 0.6094 0.4876 0.4548 Porphyromonas gingivalis strain ATCC 33277 [id: 4816]

0.4619 0.6366 0.512 0.4421 Prevotella copri strain DSM 18205 [id: 19097]

0.3855 0.6198 0.4804 0.4989 Prochlorococcus marinus MIT 9211 strain MIT9211 [id: 4822]

0.6035	0.6136	0.441	0.4099	Propionibacterium acnes TypeIA2 strain P.acn33 [id: 17263]
0.4012	0.6085	0.4785	0.4683	Proteus mirabilis strain HI4320 [id: 4841]
0.6132	0.5893	0.4675	0.4545	Pseudomonas fluorescens strain Pf0-1 [id: 7723]
0.7334	0.6055	0.4279	0.4412	Pseudonocardia dioxanivorans strain CB1190 [id: 11563]
0.3529	0.6433	0.4987	0.4767	Psychroflexus torquis strain ATCC 700755 [id: 21251]
0.67	0.5939	0.4464	0.4672	Ralstonia eutropha strain H16 [id: 3297]
0.6086	0.6154	0.4495	0.4122	Rhizobium tropici strain CIAT 899 [id: 21479]
0.6914	0.5982	0.436	0.4569	Rhodobacter sphaeroides ATCC 17029 [id: 16387]
0.6294	0.6173	0.4295	0.4419	Rhodococcus erythropolis strain CCM2595 [id: 21272]
0.5544	0.5864	0.4721	0.4682	Rhodopirellula baltica SH strain 1 [id: 3179]
0.6527	0.5555	0.4652	0.4866	Rhodothermus marinus strain DSM 4252 [id: 7742]
0.3181	0.6562	0.4954	0.4927	Rickettsia bellii strain OSU 85-389 [id: 3167]
0.6094	0.5827	0.4421	0.4765	Roseiflexus castenholzii strain DSM 13941 [id: 3150]
0.5223	0.5951	0.4775	0.4794	Shigella boydii strain Sb227 [id: 2338]
0.61	0.6341	0.4709	0.4201	Slackia heliotrinireducens strain DSM 20476 [id: 7775]
0.7212	0.5964	0.4536	0.4593	Sorangium cellulosum strain So0157-2 [id: 21337]
0.6836	0.5941	0.4453	0.4576	Sphaerobacter thermophilus strain DSM 20745 [id: 15776]
0.336	0.6508	0.4922	0.5064	Staphylococcus aureus strain 04-02981 [id: 19037]
0.3605	0.6367	0.4828	0.4672	Streptococcus agalactiae strain 2603V/R [id: 2238]
0.7234	0.5998	0.4401	0.4302	Streptomyces cf. griseus strain XylebKG-1 [id: 11326]
0.7142	0.6012	0.438	0.439	Streptosporangium roseum strain DSM 43021 [id: 8984]
0.348	0.6678	0.4909	0.4946	Sulfurimonas denitrificans DSM 1251 strain ATCC 33889 [id: 2124]

0.3918	0.6358	0.4809	0.4575	Sulfurospirillum barnesii strain SES-3 [id: 21571]
0.5027	0.582	0.472	0.4397	Synechococcus sp. strain ATCC 27264; PCC 7002 [id: 15707]
0.602	0.6043	0.4709	0.4671	Syntrophobacter fumaroxidans strain MPOB [id: 16636]
0.5454	0.5598	0.4647	0.445	Thermosynechococcus elongatus strain BP-1 [id: 18743]
0.6685	0.5726	0.4655	0.4817	Thioalkalivibrio nitratireducens strain DSM 14787 [id: 21734]
0.6641	0.5972	0.4539	0.435	Thiobacillus denitrificans strain ATCC 25259 [id: 2064]
0.5037	0.6354	0.4749	0.4338	Treponema azotonutricium strain ZAS-9 [id: 15520]
0.3752	0.6454	0.4797	0.4911	Treponema pedis strain T A4 [id: 21744]
0.538	0.6091	0.4654	0.4571	Turneriella parva strain DSM 21527 [id: 21514]
0.2615	0.6514	0.5132	0.4626	Ureaplasma urealyticum serovar 5 strain ATCC 27817 [id: 2043]
0.3983	0.647	0.4815	0.4546	Veillonella atypica strain ACS-134-V-Col7a [id: 10356]
0.4833	0.5968	0.474	0.4693	Vibrio cholerae strain BX 330286 [id: 7901]
0.3521	0.6595	0.496	0.5091	Wolbachia endosymbiont strain TRS of Brugia malayi [id: 1970]
0.4905	0.5979	0.4731	0.4708	Yersinia pestis D106004 [id: 14980]
